# Microenvironment‐Responsive Nanomedicine Enables Vertical Modulation of Mitochondrial Pathological Networks in Myocardial Ischemia–Reperfusion Injury

**DOI:** 10.1002/advs.76519

**Published:** 2026-07-13

**Authors:** Jue Wang, Jia Zhou, Wenqin Yuan, Tianjiao Zhao, Yunying Huang, Yingci Xia, Yuting Lin, Fei Li, Qiong Huang, Chong Liu, Kelong Ai, Qun Qin

**Affiliations:** ^1^ Department of Pharmacy, Xiangya Hospital Central South University Changsha China; ^2^ Xiangya School of Pharmaceutical Sciences Central South University Changsha China; ^3^ Hunan Province International Science and Technology Innovation Cooperation Base for Early Clinical Trials of Biological Agents, Xiangya Hospital Central South University Changsha China; ^4^ Department of Pharmacy Yizhang County People's Hospital Chenzhou Hunan China; ^5^ National Clinical Research Center for Geriatric Disorders, Xiangya Hospital Central South University Changsha China; ^6^ Hunan Provincial Key Laboratory of Cardiovascular Research, Xiangya School of Pharmaceutical Sciences Central South University Changsha China; ^7^ Key Laboratory of Aging‐related Bone and Joint Diseases Prevention and Treatment, Ministry of Education, Xiangya Hospital Central South University Changsha China

**Keywords:** melanin nanocapsules, microenvironment‐responsive, mitochondrial targeting, myocardial ischemia–reperfusion injury, vertical modulation

## Abstract

Myocardial ischemia–reperfusion injury (MIRI) is a severe and largely unavoidable complication of reperfusion therapy and remains a major determinant of poor outcomes after acute myocardial infarction. Here, we developed a mitochondria‐targeted, ischemic microenvironment–responsive nanotherapeutic, termed Berberine/Isoliensinine‐loaded Melanin Nanocomposite (BIM), by co‐encapsulating the anti‐inflammatory agent berberine (BBR) and the anti‐apoptotic agent isoliensinine (ILS) within a melanin capsule with a controllable channel (MCC). Owing to its appropriate size, negative surface charge, and dual responsiveness to acidic pH and reactive oxygen species, BIM preferentially accumulates in injured myocardium and targets damaged mitochondria, enabling precise drug release at the pathological core. Through a structured pharmacological design, MCC scavenges mitochondrial reactive oxygen species, BBR suppresses inflammatory signaling, and ILS inhibits intrinsic apoptosis, together achieving vertical modulation of mitochondrial pathological networks. Consequently, BIM attenuates oxidative stress, limits inflammatory amplification, reduces cardiomyocyte apoptosis, and markedly decreases infarct size. These findings establish mitochondria‐centered vertical network modulation as a precise therapeutic strategy for MIRI.

## Graphical abstract

A mitochondria‐targeted, microenvironment‐responsive nanoplatform enables pH/ROS‐triggered co‐delivery of berberine and isoliensinine to orchestrate ROS scavenging, inflammation suppression, and apoptosis inhibition. This vertical modulation of mitochondrial pathological networks preserves mitochondrial function and effectively mitigates myocardial ischemia–reperfusion injury.

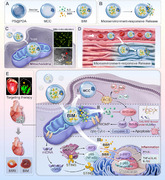

## 1 Introduction

Myocardial ischemia–reperfusion injury (MIRI) remains a major unresolved bottleneck in the treatment of acute myocardial infarction. According to the latest estimates from the global burden of cardiovascular disease, the number of individuals living with cardiovascular diseases reached 437 million by 2023, with ischemic heart disease accounting for the largest proportion of this burden [1]. Although timely revascularization markedly reduces acute mortality, reperfusion itself can trigger additional myocardial injury, thereby partially offsetting its therapeutic benefit [2]. Extensive evidence from both experimental and clinical studies indicates that reperfusion‐associated injury substantially enlarges the final infarct size and represents a key determinant of poor prognosis [3]. Against this background, achieving effective coronary reperfusion while simultaneously limiting reperfusion‐related myocardial damage has emerged as a central scientific and clinical challenge constraining further improvements in the overall outcomes of acute myocardial infarction.

Mitochondrial injury in cardiomyocytes represents a subcellular pathological core of MIRI and initiates a cascade of pathological events at the early stage of reperfusion [4]. The abrupt reintroduction of oxygen during reperfusion rapidly oxidizes the reducing equivalents accumulated in the electron transport chain during ischemia, leading to a burst of mitochondrial reactive oxygen species (mtROS) and conformational activation of stress‐sensing bcl‐2 homology domain (BH3)‐only proteins [5, 6]. Activated BH3‐only proteins interact with the pro‐apoptotic protein Bcl‐2‐associated X protein (Bax), promote its oligomerization at the mitochondrial outer membrane, and concurrently suppress the function of the anti‐apoptotic protein B‐cell lymphoma‐2 (Bcl‐2), ultimately resulting in mitochondrial outer membrane permeabilization (MOMP). This pivotal event allows cytochrome C (Cyt C) to be released into the cytosol and activates a Caspase‐3‐dependent intrinsic apoptotic program [7]. Excess mtROS further disrupts mitochondrial membrane integrity, leading to the exposure of mitochondrial deoxyribonucleic acid (mtDNA) and other danger‐associated molecular patterns (DAMPs) [8]. mtDNA is rapidly sensed by the cyclic GMP‐AMP synthase–stimulator of interferon genes (cGAS–STING) innate immune pathway, which drives the transcriptional activation of interferon regulatory factor 3 (IRF3) and nuclear factor κB (NF‐κB). IRF3 activation induces the upregulation of type I interferons such as interferon‐β (IFN‐β), whereas NF‐κB promotes the robust production of pro‐inflammatory cytokines, including tumor necrosis factor α (TNF‐α) [9]. Together, these cascades form a mitochondrial‐centered pathological network linking oxidative stress, apoptosis, and inflammation, ultimately leading to an expansion of myocardial tissue injury. Current pharmacological strategies for MIRI have limited capacity to cover this entire network, and conventional small‐molecule drugs generally lack sufficient regulatory depth to address both upstream pathogenic triggers and downstream cellular responses [2, 10]. Therefore, the development of targeted delivery systems capable of vertically modulating mitochondrial pathological networks has emerged as a critical research direction. Such systems aim to simultaneously suppress oxidative stress at the initiation stage and intervene in downstream apoptotic and inflammatory programs, thereby enabling precise and strategically layered control to overcome the therapeutic bottleneck of MIRI.

**SCHEME 1:**
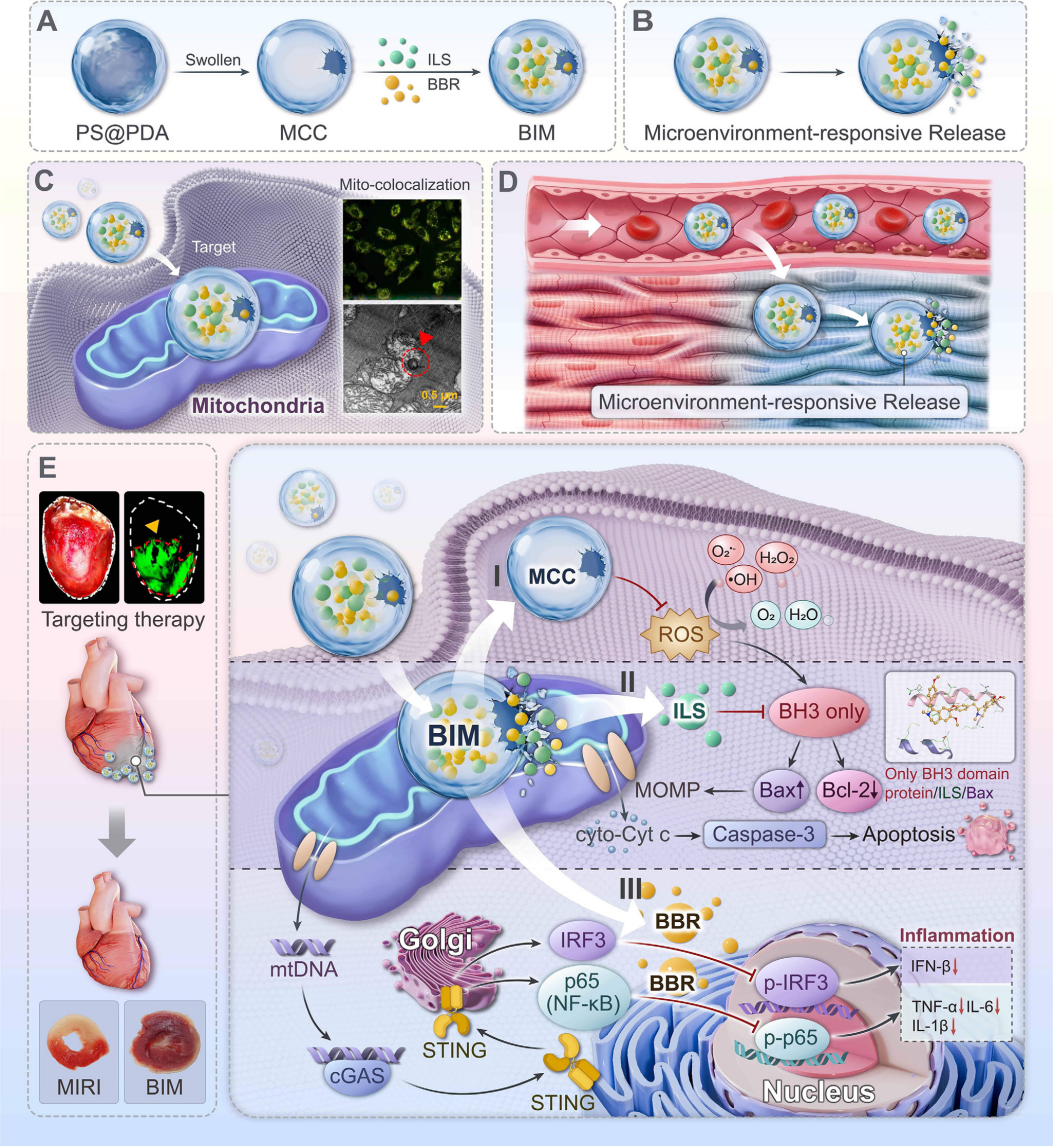
Schematic summary of BIM‐mediated therapeutic effects and underlying mechanisms on MIRI. (A) Schematic diagram illustrating the synthesis of BIM. (B) Schematic diagram illustrating the microenvironment‐responsive release property of BIM. (C) Schematic diagram illustrating the mitochondrial targeting of BIM in MIRI. (D) Schematic diagram illustrating that BIM enters the MIRI myocardium through the disrupted vascular endothelial barrier to exert therapeutic effects. (E) Schematic illustration of BIM‐mediated vertical modulation of MIRI‐associated pathological cascades. MCC provides mitochondrial targeting, drug delivery, and upstream ROS scavenging; ILS primarily inhibits mitochondrial‐dependent apoptosis; BBR exerts a major anti‐inflammatory effect. Together, these components coordinately regulate the pathological network from upstream triggers to downstream execution.

To advance strategies for vertical modulation of mitochondrial pathological networks, we developed a synergistic design that integrates polydopamine (PDA)‐based nanocarriers with natural therapeutics and constructed a berberine and isoliensinine‐loaded melanin nanocomposite (BIM) for multidimensional targeted therapy. As illustrated in Scheme 1A, this system employs MCC as the carrier, enabling the co‐loading of the natural compounds isoliensinine (ILS) and berberine (BBR). This design yields a targeted nanocomposite, BIM, with a structured framework for vertical modulation. BIM exhibits release behavior that is responsive to the ischemic myocardial microenvironment. First, in the mildly acidic conditions of ischemic regions caused by lactate accumulation (pH 6.0), the PDA framework undergoes conformational changes that markedly accelerate drug release compared with neutral conditions (pH 7.4). Second, excessive mtROS in the vicinity of mitochondria within ischemic tissue triggers the redox responsiveness of PDA, allowing precise localization to sites of mitochondrial injury and enabling on‐demand release of therapeutic agents directly at damaged myocardial mitochondria (Scheme 1B–D). As a synergistic system composed of MCC, ILS, and BBR, BIM enables vertical modulation of the key pathological processes underlying MIRI. As depicted in Scheme 1E, MCC exhibits a strong capacity for reactive oxygen species (ROS) scavenging and directly targets the initial stage of mitochondrial injury. ILS stably binds to the BH3 domain of pro‐apoptotic proteins, thereby preventing Bax activation, reducing the occurrence of MOMP, and suppressing downstream apoptosis driven by Cyt C release. In parallel, BBR inhibits the nuclear translocation and activation of IRF3 and NF‐κB, leading to reduced production of type I interferons and pro‐inflammatory cytokines and effectively restraining downstream inflammatory responses. Through this structured design, BIM achieves coordinated control from microenvironment‐responsive drug release to multi‐level and multi‐dimensional therapeutic intervention, providing a new paradigm for the precise treatment of MIRI.

## 2 Results

### 2.1 Preparation and Characterization of BIM

**FIGURE 1:**
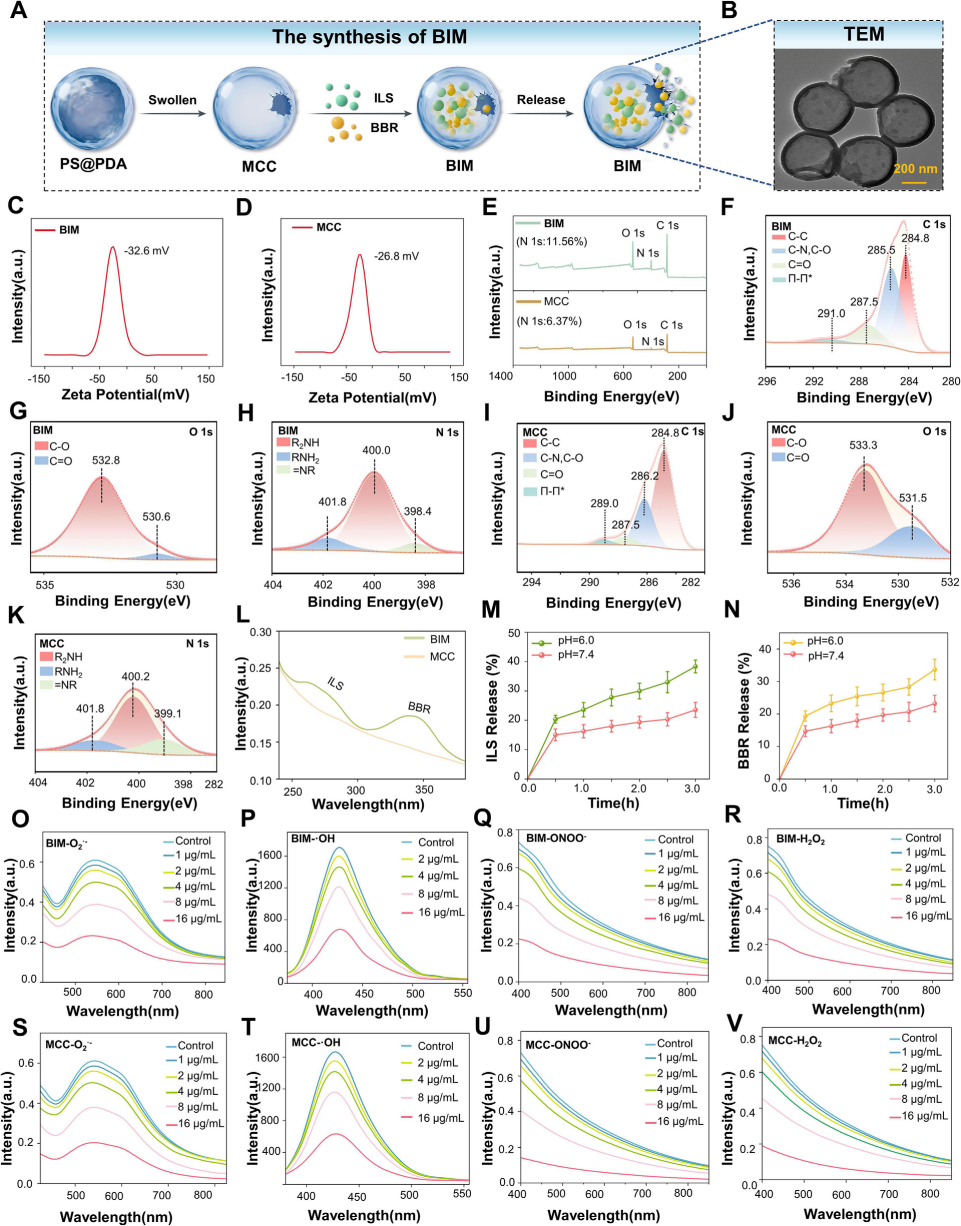
Preparation and characterization of BIM. (A) Schematic diagram illustrating the synthesis of BIM. (B) TEM image of BIM, scale bar: 200 nm. (C) Zeta potential map of BIM. (D) Zeta potential map of MCC. (E) XPS spectrograms of BIM and MCC. (F–H) XPS spectra of C 1s, N 1s, and O 1s in BIM. (I–K) XPS spectra of C 1s, N 1s, and O 1s in MCC. (L) Loading curves of ILS and BBR. (M,N) Cumulative drug release from ILS and BBR in dispersed system fractions at pH 6.0 and 7.4. (O–R) O_2_·^−^, ·OH, ONOO^−^, H_2_O_2_ scavenging capacity of BIM. (S–V) O_2_·^−^, ·OH, ONOO^−^, H_2_O_2_ scavenging capacity of MCC.

In this study, polystyrene microspheres (PS) were used as templates and dopamine hydrochloride (DA) as the monomer to form a PDA (artificial melanin) coating through multilayer deposition on the microsphere surface, followed by spontaneous oxidative polymerization. Subsequently, the PS core was removed by emulsification‐induced pore formation and tetrahydrofuran extraction, yielding the MCC. ILS and BBR were then loaded into MCC to generate a dual‐drug nanocomposite, termed BIM (Figure 1A). Transmission electron microscopy (TEM) images (Figure 1B and Figure S1) showed that both BIM and MCC exhibit hollow vesicle‐like structures with an open pore, an average diameter of approximately 300 nm, and a capsule‐like morphology, indicating a favorable capacity for drug loading. Zeta potential measurements (Figure 1C,D) revealed that both BIM and MCC possess strongly negative surface charges, suggesting electrostatic repulsion from negatively charged plasma proteins in vivo, which is advantageous for prolonging systemic circulation. X‐ray photoelectron spectroscopy (XPS) further characterized the elemental composition and chemical features of BIM and MCC. Both materials contained carbon, nitrogen, and oxygen, with carbon as the dominant element (BIM, 50.7%; MCC, 54.4%). Notably, the nitrogen content was substantially higher in BIM (11.6%) than in MCC (6.4%), likely reflecting the contribution of ILS and BBR loading to the increased nitrogen proportion (Figure 1E). Analysis of the high‐resolution C 1s XPS spectra (Figure 1F,I) revealed that both BIM and MCC exhibit distinct C─O and C═O peaks, indicating the presence of abundant carboxyl and phenolic hydroxyl groups, which account for their overall negative surface charge. The O 1s high‐resolution spectra (Figure 1G,J) further showed that, compared with MCC, BIM displays a higher relative abundance of C─O bonds. This difference is likely attributable to the successful incorporation of ILS, which contains multiple hydroxyl oxygen (C─OH) groups and ether (R–O–R′) linkages in its molecular structure. Consistently, the N 1s high‐resolution spectra (Figure 1H,K) demonstrated a markedly higher proportion of secondary amine nitrogen in BIM than in MCC. This increase is most plausibly explained by the loading of BBR, as the alkaline conditions used throughout dopamine polymerization favor the presence of nitrogen in BBR predominantly in the form of secondary amines. UV–vis absorption spectra (Figure 1L) further confirmed drug loading, as BIM exhibited characteristic absorption peaks of ILS at approximately 280 nm and of BBR at approximately 352 nm that were absent in MCC, indicating the successful incorporation of both compounds into the MCC framework. In addition, the drug loading efficiency was quantified by ultraviolet spectrophotometry using the equation: loading efficiency = [(total mass of ILS and BBR added − total mass of ILS and BBR in the supernatant) / total mass of BIM] × 100%. The loading efficiency was consistently maintained at approximately 78.0%. These results collectively demonstrate that MCC can successfully and stably encapsulate both ILS and BBR.

BIM exhibited sustained release of BBR and ILS under both neutral (pH 7.4) and mildly acidic (pH 6.0) conditions. At 3 h, BIM released approximately 33.6% of BBR and 38.3% of ILS in the acidic environment, whereas under neutral conditions, the cumulative release of both BBR and ILS was about 23.0% over the same period (Figure 1M,N). These findings indicate that BIM displays a release profile responsive to the mildly acidic microenvironment characteristic of ischemic tissue. As shown in Figure 1O–R, BIM efficiently scavenged pathological ROS, including O_2_·^−^, ·OH, H_2_O_2_, and ONOO^−^, and exhibited a clear dose‐dependent antioxidant capacity. Notably, Figure 1S–V shows that the ROS‐scavenging activity of the empty carrier MCC was more pronounced than that of BIM, which may be attributed to the reduced exposed surface area of PDA after loading BBR and ILS. Overall, these results demonstrate that BIM successfully incorporates the two natural compounds BBR and ILS and retains the ability to eliminate ROS in the acidic ischemic microenvironment.

### 2.2 Evaluation of BIM Biodistribution and Therapeutic Effect

**FIGURE 2:**
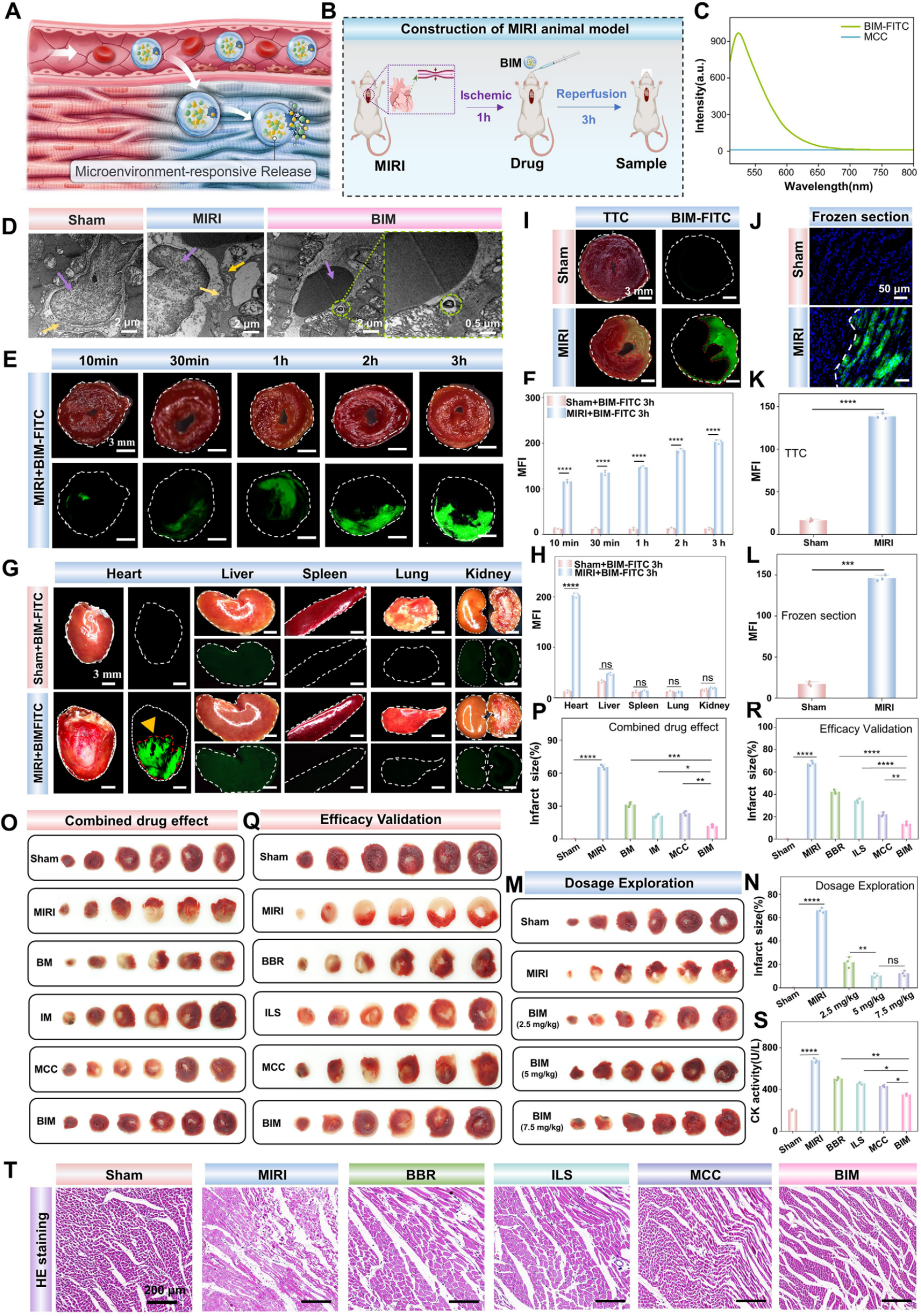
Evaluation of BIM biodistribution and therapeutic effect. (A) Schematic diagram illustrating that BIM enters the MIRI myocardium via microenvironment‐responsive release. (B) Schematic diagram illustrating the establishment of the MIRI model in SD rats. (C) Fluorescence signal of FITC‐labeled BIM. (D) Representative TEM images of vessels from Sham, MIRI, and BIM‐treated rats with BIM localized to myocardial mitochondria in the infarct zone (yellow arrow: endothelial junction; violet arrow: vascular lumen; green circle: BIM). (E) Representative images of BIM‐FITC fluorescence imaging and bright field images of the heart at 10, 30 min, 1, 2, and 3 h after MIRI rats; scale bar: 3 mm; and (F) fluorescence intensity statistics. (G) Representative images of BIM‐FITC fluorescence imaging and bright field of the heart, liver, spleen, lungs, and kidneys of Sham and MIRI rats 3 h after BIM‐FITC administration and (H) quantitative analysis, scale bar: 3 mm. (I) Representative images of TTC staining of TTC hearts 3 h after BIM‐FITC injection and (K) quantitative analysis, scale bar: 3 mm. (J) Representative fluorescence imaging of frozen sections of rat hearts from Sham and MIRI groups and (L) quantitative analysis, scale bar: 50 µm. MFI: mean fluorescence intensity. (M) TTC staining of heart tissues to explore the dose of drugs administered to BIM animals and (N) infarct size statistics. (O) TTC staining of heart tissue to validate the efficacy of combined drug therapy and (P) infarct size statistics. (Q) TTC staining of heart tissue to verify the efficacy of BIM and (R) infarct size statistics. Data are expressed as mean ± SE. Statistical significance was analyzed by one‐way ANOVA using the Tukey post hoc test. (*n* = 3, ^*^ *p* < 0.05, ^**^ *p* < 0.01, ^***^ *p* < 0.001, ^****^ *p* < 0.0001, ns: *p* > 0.05).

MIRI exacerbates myocardial vascular endothelial injury and disrupts the integrity of inter‐endothelial junctions, leading to further enlargement of endothelial gaps and thereby creating favorable conditions for the local accumulation of BIM [11]. Owing to its appropriate particle size, BIM may pass through these enlarged vascular gaps in MIRI tissue and subsequently respond to ROS enriched in the acidic microenvironment, enabling microenvironment‐responsive drug release and targeted therapy (Figure 2A). To support this hypothesis, a rat MIRI model was established (Figure 2B). Male Sprague–Dawley rats from the same litter (8 weeks old, 220–250 g) underwent ligation of the left anterior descending coronary artery for 1 h. Drugs were administered intravenously at the onset of reperfusion, with the Sham and MIRI groups receiving equal volumes of vehicle. After 3 h of reperfusion, hearts were harvested for analysis. TEM was used to examine the vascular endothelial status in myocardial tissue (Figure 2D). In the Sham group, the vascular basement membrane remained intact and endothelial junctions were well preserved. In contrast, the MIRI group exhibited markedly enlarged capillary gaps and disrupted endothelial junctions, providing potential access sites for BIM accumulation. In the BIM‐treated group, particulate structures with diameters of approximately 300 nm were observed around myocardial vessels, providing initial evidence that BIM can traverse and be retained within ischemia–reperfusion–injured myocardial tissue. BIM achieves selective passive targeting of MIRI lesions, thereby reducing systemic exposure to BBR and ILS and potentially limiting off‐target toxicity in other organs.

To visualize the in vivo distribution of BIM more directly, BIM was labeled with fluorescein isothiocyanate (FITC), a strong fluorescent probe, to generate BIM‐FITC (Figure 2C). 5 min before reperfusion, BIM‐FITC was administered via the sublingual vein. Hearts, livers, spleens, lungs, and kidneys were collected at 10, 30 min, 1, 2, and 3 h after injection, and the biodistribution of BIM‐FITC was examined using a stereofluorescence microscope. As shown in Figure 2E,F and Figure S2, BIM‐FITC accumulation in heart sections from the infarct region increased over time. Compared with the Sham group, fluorescence intensities in the MIRI group were approximately 4.0‐, 5.5‐, 6.3‐, 7.0‐, and 7.6‐fold higher at 10, 30 min, 1, 2, and 3 h after administration, respectively. These results indicate that BIM rapidly targets the injured myocardium, with progressive signal enhancement and pronounced accumulation in the infarct area by 3 h. In contrast, fluorescence signals in other organs were markedly lower than those observed in the MIRI heart, supporting the preferential localization of BIM to ischemia–reperfusion–injured myocardial tissue. In addition, in both the Sham and MIRI groups, the fluorescence intensity of BIM‐FITC in the liver increased progressively over time. In MIRI rats, the hepatic fluorescence signal at 3 h was approximately 2.1‐fold higher than that at 10 min, indicating that BIM is primarily metabolized through the liver (Figure 2G,H and Figure S3). After triphenyltetrazolium chloride (TTC) staining, heart sections showed a high spatial overlap between the myocardial infarct area and regions of BIM‐FITC signal enrichment (Figure 2I,K). Consistently, frozen heart sections further demonstrated pronounced accumulation of BIM‐FITC in the infarcted myocardium. Compared with the Sham group, fluorescence intensity in the MIRI group was increased by approximately 8.4‐fold (Figure 2J,L). Collectively, these stereofluorescence observations indicate that BIM rapidly and selectively targets the MIRI heart, is stably retained within the infarct region, does not accumulate in healthy myocardium, and undergoes normal hepatic metabolism. These features together suggest that BIM possesses favorable biocompatibility in vivo.

In the MIRI animal model, the optimal therapeutic dose of BIM was first determined by assessing post‐treatment infarct size. TTC staining revealed that, compared with 2.5 mg/kg, administration of 5 mg/kg BIM produced a more pronounced reduction in infarct area, with no significant difference observed between the 5 and 7.5 mg/kg doses (Figure 2M,N). To further evaluate the therapeutic efficacy of BIM in MIRI, additional groups were included, comprising BMC (MCC loaded with BBR alone), IMC (MCC loaded with ILS alone), and MCC, all administered at a uniform dose of 5 mg/kg. The effects of the three major components were assessed by TTC staining. The results showed that BIM achieved the most substantial reduction in infarct size compared with the BMC, IMC, and MCC groups, lowering the infarct area to below 15% (Figure 2O,P). After establishing the optimal dose for nanotherapy in MIRI and confirming that co‐loading BBR and ILS within MCC conferred superior efficacy compared with single‐drug loading, six groups were ultimately defined for therapeutic evaluation: Sham, MIRI, BBR (5 mg/kg), ILS (5 mg/kg), MCC (5 mg/kg), and BIM (5 mg/kg). TTC staining results (Figure 2Q,R) consistently demonstrated the robust efficacy of BIM, with the mean infarct size in the MIRI group reaching 67.9%, which was markedly reduced to approximately 13.7% following BIM treatment, indicating effective reversal of MIRI‐associated infarct expansion. Notably, the therapeutic effects of the other treatment groups were clearly inferior to those of BIM. Serum creatine kinase (CK) measurements (Figure 2S) showed a marked elevation of CK levels in the MIRI group, whereas treatment with BBR, ILS, MCC, and BIM all reduced serum CK to varying extents. Among these, BIM produced the most pronounced reduction, indicating a superior capacity to attenuate myocardial injury. In addition, hematoxylin and eosin staining revealed extensive myofibrillar disruption, dissolution, and disorganized alignment in the MIRI group. In contrast, BIM treatment markedly improved myocardial tissue architecture, with tightly arranged and intact myocardial fibers and well‐preserved nuclei observed at the 5 mg/kg dose (Figure 2T). To directly assess the effect of drug treatment on cardiac function in rats, the reperfusion period was extended to 24 h, and echocardiography was performed to measure left ventricular ejection fraction (LVEF) and left ventricular fractional shortening (LVFS) across different groups. The results showed that, at 24 h post‐MIRI, LVEF and LVFS in the MIRI group were markedly reduced to 36.9% and 18.2%, respectively, corresponding to 40.5% and 28.5% relative to the Sham group. In the BIM group, LVEF and LVFS were significantly restored to 78.4% and 48.2%, respectively, corresponding to 86.4% and 75.5% relative to the Sham group, indicating that BIM treatment effectively preserved and improved cardiac function. Treatment with BBR, ILS, or the MCC carrier alone also provided varying degrees of functional protection, but the effect was markedly lower than that observed with BIM (Figure S4). Together, these findings demonstrate that BIM effectively rescues the myocardium from MIRI and that the composite system composed of MCC, ILS, and BBR exerts a synergistic therapeutic effect.

### 2.3 Therapeutic Mechanism of BIM on MIRI

**FIGURE 3:**
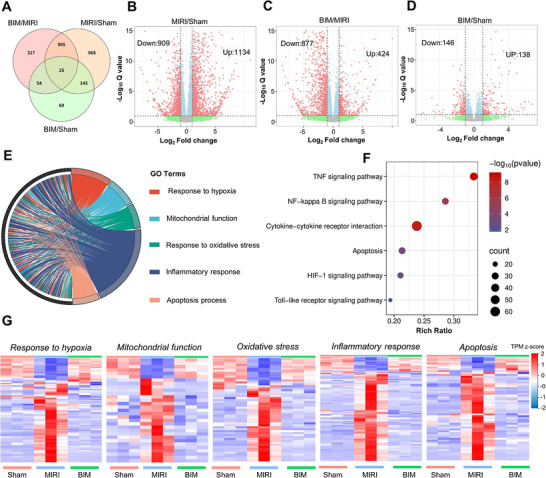
Therapeutic mechanism of BIM on MIRI. (A) VENN/UpSetR graph analysis showing DEGs between different comparison groups. (B) Volcano plot depicting DEGs between cardiac tissues of MIRI vs Sham, highlighting 1134 up‐regulated genes and 909 down‐regulated genes. (C) Volcano plot depicting DEGs between cardiac tissues of BIM vs MIRI, highlighting 424 up‐regulated genes and 877 down‐regulated genes. (D) Volcano plot depicting DEGs between cardiac tissues of BIM vs Sham treatment groups, highlighting 138 up‐regulated genes and 146 down‐regulated genes. (E) GO enrichment analysis of biological processes specifically involved in DEGs between the MIRI and BIM treatment groups. (F) KEGG pathway enrichment analysis of DEGs between MIRI and BIM treatment groups. (G) Heatmap showing DEGs between Sham, MIRI, and BIM treatment groups in pathways identified by GO enrichment analysis. Data are expressed as mean ± SE. Statistical significance was analyzed by one‐way ANOVA using the Tukey post hoc test. (*n* = 3, ^*^ *p* < 0.05, ^**^ *p* < 0.01, ^****^ *p* < 0.0001).

To further elucidate the therapeutic mechanism of BIM in MIRI, transcriptomic profiling (RNA‐Seq) was performed on cardiac tissues from Sham, MIRI, and BIM‐treated rats to characterize gene expression changes at the RNA level. Differential expression analysis was first summarized using a Venn diagram. As shown in Figure 3A, 2043 differentially expressed genes (DEGs) were identified between the MIRI and Sham groups, whereas 1301 DEGs were detected between the BIM and MIRI groups. In contrast, only 284 DEGs were observed between the BIM and Sham groups, suggesting that BIM treatment largely restores the transcriptomic profile toward a near‐physiological state. Volcano plot analysis further revealed the direction and magnitude of transcriptional alterations. Compared with the Sham group, the MIRI group exhibited 1134 upregulated and 909 downregulated genes, reflecting extensive pathological transcriptional reprogramming induced by ischemia–reperfusion. In comparison with the MIRI group, BIM treatment resulted in 424 upregulated and 877 downregulated genes, indicating a predominant suppression of MIRI‐induced pathological gene activation. Notably, relative to the Sham group, the BIM group showed only modest transcriptional changes, with 138 genes upregulated and 146 downregulated (Figure 3B–D), further supporting the notion that BIM mitigates aberrant gene expression rather than introducing new transcriptional disturbances. The 1301 DEGs between the BIM and MIRI groups were subsequently subjected to functional enrichment and clustering analyses. Gene Ontology (GO) analysis demonstrated that BIM treatment primarily affected biological processes closely associated with MIRI pathogenesis, including response to hypoxia, mitochondrial function, response to oxidative stress, inflammatory response, and apoptotic processes. Chord diagram visualization highlighted extensive overlap among genes involved in these biological processes, suggesting a tightly interconnected regulatory network rather than isolated pathways (Figure 3E). This finding is consistent with the multifactorial nature of MIRI and supports the concept that effective intervention requires coordinated modulation across multiple pathological axes. Kyoto Encyclopedia of Genes and Genomes (KEGG) pathway analysis further identified key signaling pathways modulated by BIM. As shown in Figure 3F, DEGs between the MIRI and BIM groups were significantly enriched in apoptosis‐ and inflammation‐related pathways, including the apoptosis pathway, TNF signaling pathway, NF‐κB signaling pathway, and cytokine–cytokine receptor interaction. These results indicate that BIM effectively suppresses transcriptional programs associated with cardiomyocyte apoptosis and inflammatory signaling triggered by ischemia–reperfusion. Importantly, while apoptosis and inflammation represent direct effector pathways of myocardial injury, hypoxia response and mitochondrial‐related functions appeared as upstream enriched processes, suggesting that mitochondrial dysfunction and hypoxic stress may act as initiating events that drive downstream inflammatory and apoptotic cascades. This hierarchical pattern supports the hypothesis that BIM exerts its therapeutic effects by modulating key crosstalk nodes linking mitochondrial stress to inflammatory and apoptotic signaling. Hierarchical clustering heatmaps further illustrated global expression patterns of enriched genes across experimental groups. The transcriptomic profile of the BIM group closely resembled that of the Sham group, while showing clear separation from the MIRI group (Figure 3G). This pattern suggests that BIM not only suppresses pathological gene expression induced by MIRI but also restores the expression of genes associated with mitochondrial homeostasis, redox balance, and cellular survival. Collectively, these genes are predominantly involved in hypoxia response, mitochondrial function, oxidative stress regulation, inflammation, and apoptosis. Based on these transcriptomic findings, we propose that BIM confers cardioprotection by coordinately regulating interconnected pathological processes at the transcriptional level. These results provide a systems‐level rationale for the observed functional benefits of BIM and form the basis for subsequent in vivo and in vitro experiments aimed at validating the identified molecular pathways and regulatory mechanisms.

### 2.4 BIM Attenuates MIRI‐Induced Mitochondrial Damage

**FIGURE 4:**
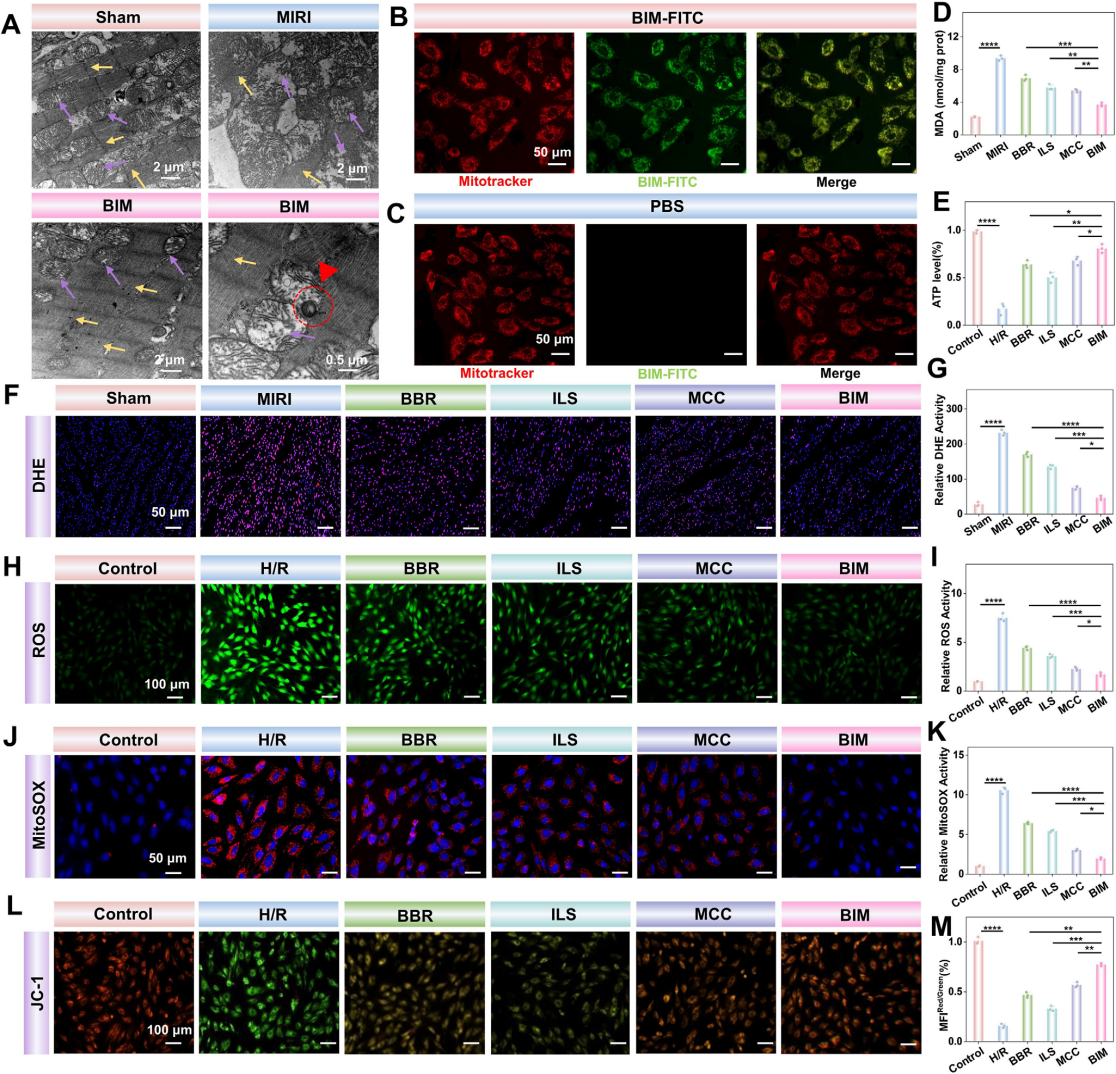
BIM attenuates MIRI‐induced mitochondrial damage. (A) TEM images of mitochondria in cardiac tissues of Sham, MIRI, and BIM‐treated groups. (B,C) Co‐localization of BIM‐FITC or PBS with H9c2 cell mitochondria for fluorescent staining; scale bars: 2 and 0.5 µm. (D) Expression levels of MDA in different groups. (E) ATP production by H9c2 cells in different groups. (F) DHE staining of heart tissue and (G) quantitative analysis; scale bar: 50 µm. (H) Fluorescence staining of ROS in H9c2 cells in different groups and (I) quantitative analysis; scale bar: 100 µm. (J) Fluorescent staining of MitoSOX red color in different groups of H9c2 cells and (K) quantitative analysis; scale bar: 50 µm. (L) Fluorescent staining of JC‐1 in H9c2 cells of different groups and (M) quantitative analysis; scale bar: 100 µm. Data are expressed as mean ± SE. Statistical significance was analyzed by one‐way ANOVA using the Tukey post hoc test. (*n* = 3, ^*^ *p* < 0.01, ^**^ *p* < 0.05, ^***^ *p* < 0.001, ^****^ *p* < 0.0001).

mtROS represents one of the earliest and most central pathological drivers of MIRI. Suppressing mtROS generation can interrupt the cascade at an upstream level of the pathological network and thereby effectively improve post‐reperfusion cardiac functional recovery [12]. TEM was first used to examine myocardial fibers and mitochondrial ultrastructure in each group. In the MIRI group, myocardial fibers were severely disorganized with prominent fragmentation, indicating extensive myocardial damage (Figure 4A). Quantitative analysis of mitochondrial ultrastructure was performed using ImageJ. Compared with the Sham group, MIRI significantly reduced mitochondrial number per field and cristae number per mitochondrion, while markedly increasing mitochondrial area, indicating severe mitochondrial swelling and structural disruption. BIM treatment significantly restored mitochondrial number and cristae density while reducing mitochondrial swelling, suggesting preservation of mitochondrial structural integrity (Figure S5). Notably, BIM particles were directly observed in close proximity to mitochondria by TEM.

Unlike classical mitochondrial‐targeting ligands, BIM may achieve mitochondrial localization through interactions with proteins on the mitochondrial outer membrane (MOM). To validate this hypothesis, magnetic Fe_3_O_4_@MCC nanoparticles were synthesized by coating Fe_3_O_4_ cores with PDA. Fe_3_O_4_ nanoparticles are well known for their excellent magnetic properties and biocompatibility. The PDA surface functionalization enhances nanoparticle stability, prevents aggregation and sedimentation, and preserves magnetic responsiveness, thereby enabling convenient separation using an external magnetic field. Mitochondria were isolated from H9c2 cells and subsequently lysed to obtain mitochondrial protein extracts. The Fe_3_O_4_@MCC nanoparticles were incubated with these protein extracts, followed by magnetic separation to isolate nanoparticle–protein complexes. The adsorbed proteins were then analyzed to identify mitochondrial proteins specifically associated with MCC. Western blot (WB) analysis confirmed these interactions and demonstrated the binding affinity of MCC toward mitochondrial outer membrane proteins (Figure S6A). Among MOM proteins, voltage‐dependent anion channels (VDAC) and translocase of the outer membrane (TOM) complexes are the most abundant, collectively occupying nearly half of the mitochondrial surface area, suggesting that they are likely primary binding sites for MCC [13]. Accordingly, the levels of VDAC1/2, TOM7, TOM20, TOM34, TOM40, and TOM70 in the magnetic precipitates were examined by WB. The results showed that, compared with Fe_3_O_4_ alone, Fe_3_O_4_@MCC exhibited stronger binding to TOM7, TOM20, TOM34, VDAC1/2, TOM40, and TOM70, confirming the high affinity of MCC for these MOM proteins (Figure S6B). To further confirm the mitochondrial targeting capacity of BIM, H9c2 cells were incubated with BIM‐FITC or PBS for 24 h, followed by mitochondrial labeling with a Mitotracker probe. Compared with the PBS group, the green fluorescence of BIM‐FITC showed strong spatial colocalization with the red fluorescence of mitochondria. Quantitative analysis revealed that the Pearson correlation coefficient in the BIM‐FITC group was 5.7‐fold higher than that in the PBS group (Figure 4B,C and Figure S7). Consistent with these observations, compared with the MIRI group, malondialdehyde (MDA) levels were reduced to 74.1%, 61.8%, 57.8%, and 39.4% following treatment with BBR, ILS, MCC, and BIM, respectively (Figure 4D). These results indicate that BIM most effectively suppresses oxidative stress–induced lipid peroxidation and preserves mitochondrial membrane integrity. These in vivo results demonstrate that BIM possesses the capacity to selectively alleviate mitochondrial injury in cardiomyocytes under MIRI conditions.

To further and more comprehensively evaluate the ability of BIM to eliminate mtROS and mitigate mitochondrial damage, an H9c2 cardiomyocyte hypoxia/reoxygenation (H/R) model was established. The optimal in vitro concentration of BIM was first determined to be 5 µg/mL using the CCK‐8 assay (Figure S8). As shown in Figure 4E, ATP production in the H/R group was markedly reduced to only 17.7% of the Control level, indicating severe mitochondrial dysfunction. BIM treatment significantly restored ATP production to 81.7%. At the same concentration, ATP levels in the BBR, ILS, and MCC groups reached 64.8%, 50.9%, and 68.9% of the Control level, respectively. Intracellular superoxide levels were assessed using the dihydroethidium (DHE) probe. In vivo, myocardial ROS levels in the MIRI group were 8.4‐fold higher than those in the Sham group, indicating a pronounced oxidative burst in ischemia–reperfusion–injured myocardium. Following intravenous administration of BBR, ILS, MCC, or BIM, ROS levels were reduced to 73.5%, 58.5%, 32.4%, and 20.0% of the MIRI group, respectively (Figure 4F,G). Consistently, DCFH‐DA staining in vitro confirmed that ROS levels in the H/R group were significantly elevated, reaching 7.6‐fold those of the Control group. Treatment with BBR, ILS, MCC, and BIM reduced intracellular ROS levels to 58.8%, 47.9%, 30.3%, and 22.8%, respectively (Figure 4H,I). Together, both in vivo and in vitro findings consistently demonstrate that the BIM composite system exhibits the most potent ROS‐scavenging capability among all tested treatments. MitoSOX staining was used to specifically label mtROS. The results showed that mtROS levels in the H/R group were 10.5‐fold higher than those in the Control group. Following pharmacological intervention, BBR, ILS, MCC, and BIM each suppressed mtROS to different extents, reducing mtROS levels to 61.0%, 51.6%, 28.7%, and 18.6% of the H/R group, respectively (Figure 4J,K). Notably, BIM effectively eliminated mtROS, while the performance of the empty carrier MCC was comparable to that of BIM, indicating that MCC constitutes the key antioxidant component within the BIM composite system. Mitochondrial membrane potential was assessed using 5,5′,6,6′‐tetrachloro‐1,1′,3,3′‐tetraethylbenzimidazolylcarbocyanine iodide (JC‐1) staining. As shown in Figure 4L,M, compared with the Control group, the MFI_red/green_ (%) in the H/R group decreased from 100% to 15.7%. Treatment with BBR, ILS, MCC, and BIM increased this ratio to 46.2%, 32.6%, 56.1%, and 76.4%, respectively. These JC‐1 results clearly demonstrate the ability of BIM to restore mitochondrial membrane potential, reflecting its efficacy in alleviating mitochondrial injury and preserving mitochondrial function. To verify the protective effects of the treatment on mitochondrial function, the protein expression levels of the ATP synthase subunit ATP5A, carnitine palmitoyltransferase 1 (CPT‐1), and citrate synthase (CS) were evaluated in both in vivo and in vitro experiments. These proteins represent key enzymes involved in oxidative phosphorylation, fatty acid β‐oxidation, and the tricarboxylic acid cycle, respectively, and collectively reflect the overall metabolic capacity of mitochondria. In the in vivo study, compared with the Sham group, the protein levels of these three mitochondrial functional markers were significantly reduced in the MIRI group. BIM treatment markedly restored the expression of ATP5A, CPT‐1, and CS, showing superior effects compared with BBR, ILS, and MCC (Figure S9). Similar trends were also observed in the in vitro experiments (Figure S10). Taken together with the RNA‐seq data, these results indicate that BIM exerts a significant protective effect on mitochondrial function at both the transcriptional and translational levels. MCC serves as a safe and efficient nanocarrier that not only enables the loading and precise delivery of sufficient amounts of BBR and ILS to injured myocardium, but also possesses intrinsic capacity to scavenge mtROS. This property initiates the vertical modulation of the MIRI pathological network by BIM at the level of the primary pathological trigger.

### 2.5 BIM Vertically Modulates Intrinsic Apoptosis

**FIGURE 5:**
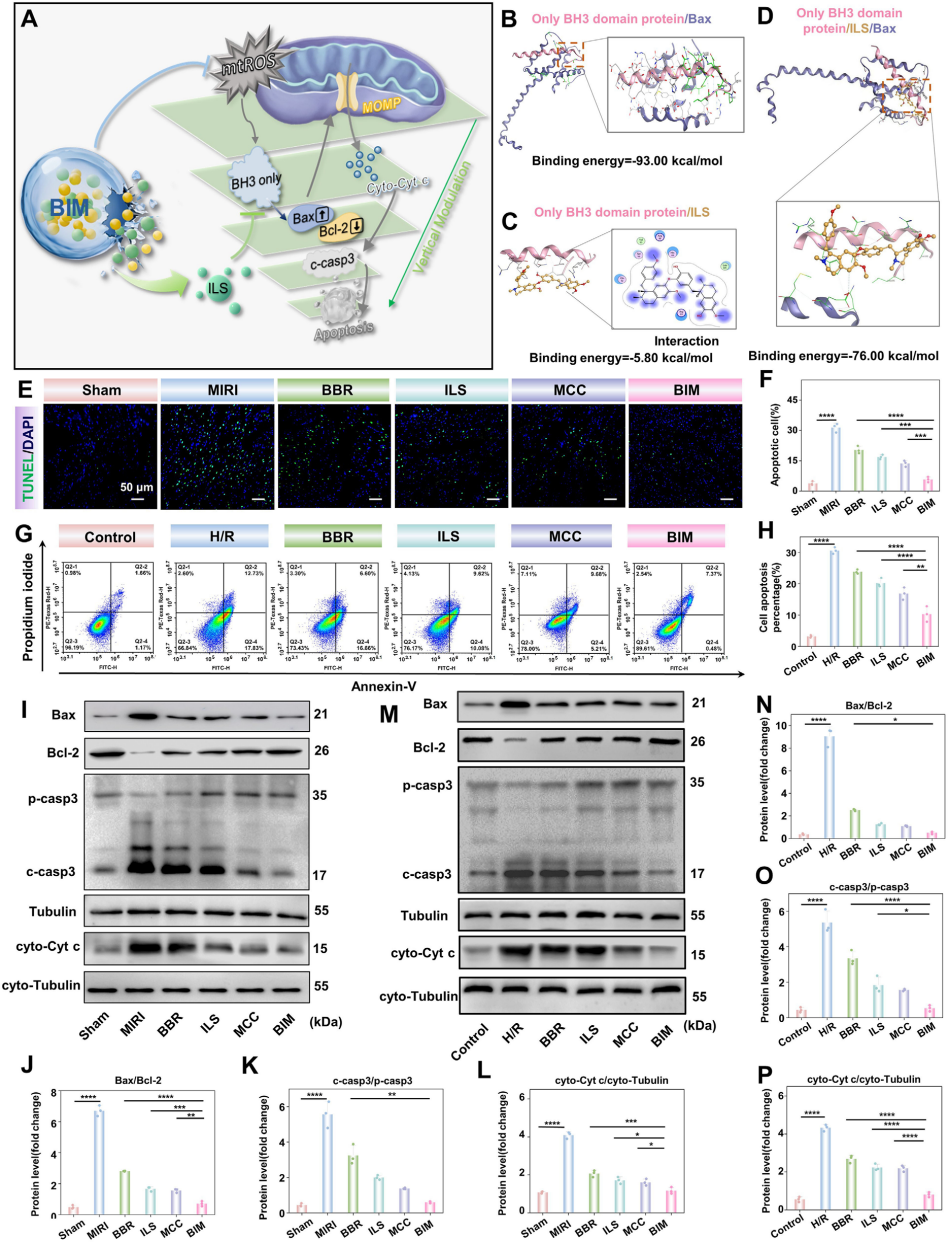
BIM vertically modulates intrinsic apoptosis. (A) Schematic representation of BIM vertically modulates intrinsic apoptosis. (B–D) Binding energy of Bax to BH3‐only domain protein, ILS to BH3‐only domain protein, ILS to Bax and BH3‐only domain protein was determined by molecular docking. (E) TUNEL staining of cardiac tissue sections from different groups and (F) quantitative analysis. Scale bar: 50 µm. (G) Flow cytometry results of anneninV‐FITC/PI in H9c2 cells under different treatment conditions and (H) quantitative analysis. (I–L) Representative images and quantitative analysis of WB of apoptosis‐related proteins in cardiac tissues, including Bax, Bcl‐2, p‐casp3, c‐casp3 and cyto‐Cyt c. (O,P) Representative images and quantitative analysis of WB of apoptosis‐related proteins in H9c2 cells, including Bax, Bcl‐2, p‐casp3, c‐casp3 and cyto‐Cyt c. Data were expressed as mean ± SE. Statistical significance was analyzed by one‐way ANOVA using the Tukey post hoc test. (*n* = 3, ^*^ *p* < 0.01, ^**^ *p* < 0.05, ^****^ *p* < 0.0001).

During ischemia–reperfusion, mtROS accumulation promotes activation and outer membrane oligomerization of Bax driven by BH3‐only proteins, leading to increased MOMP, Cyt C release, and amplification of intrinsic apoptosis through cleavage of full‐length Caspase‐3 (pro‐Caspase‐3, p‐casp3) into its activated form, cleaved Caspase‐3 (c‐casp3) [14]. BIM exerts dual actions by scavenging upstream mtROS through MCC and, in parallel, suppressing BH3‐only protein–mediated Bax activation via the controlled release of ILS. Through this coordinated strategy, BIM achieves vertical modulation at both the initiation and execution stages of the pathological cascade, thereby globally attenuating mitochondria‐dependent intrinsic apoptosis (Figure 5A). Effective interruption of the mtROS–Bax–Cyt C–c‐casp3 axis is therefore critical for the protective effects of BIM in MIRI. To examine this mechanism, molecular docking was first performed to assess the interaction between Bax and the BH3 domain, yielding a binding energy of −93.0 kcal/mol, indicative of a highly stable complex. Docking of ILS with the BH3 domain resulted in a binding energy of −5.8 kcal/mol, suggesting a direct interaction between ILS and the BH3 domain. When the BH3 domain, Bax, and ILS were docked together, the overall binding energy increased to −76.0 kcal/mol, demonstrating that ILS interferes with and weakens the stability of the BH3 domain–Bax interaction (Figure 5B–D). Apoptotic responses were next evaluated in both in vivo and in vitro settings. Terminal deoxynucleotidyl transferase–mediated dUTP nick‐end labeling (TUNEL) staining was used to quantify apoptosis in myocardial tissue from different groups. As shown in Figure 5E,F, the apoptotic rate in the MIRI group reached 31.5%, whereas BIM treatment reduced this value to 5.7%. At the same dose, myocardial apoptosis was reduced to 20.4%, 16.9%, and 13.7% in the BBR, ILS, and MCC groups, respectively. Flow cytometric analysis using Annexin V and propidium iodide dual staining further demonstrated that BIM was markedly more effective than BBR, ILS, or MCC in attenuating H/R‐induced apoptosis in vitro (Figure 5G,H). Consistently, CCK‐8 assays showed that BIM most effectively restored cell viability following H/R injury (Figure S11). WB analysis of intrinsic apoptosis–related proteins is shown in Figure 5I–L and (Figure S12. Compared with the Sham group, the MIRI group exhibited significantly elevated levels of cytosolic Cyt C (cyto‐Cyt c), and the ratio of Bax/Bcl‐2 and c‐casp3/p‐casp3. BIM treatment suppressed the level of cyto‐Cyt c, and the ratio of Bax/Bcl‐2 and c‐casp3/p‐casp3, with effects that were significantly stronger than those observed in the BBR, ILS, and MCC groups. Similar trends were observed in vitro in H9c2 cells, where WB analyses showed high concordance with the in vivo findings (Figure 5M–P and Figure S13). Notably, ILS exerted a stronger anti‐apoptotic effect than BBR, suggesting that among the two bioactive components loaded in BIM, ILS makes the dominant contribution to apoptosis inhibition. By actively scavenging ROS to control the initiation of the pathological cascade and releasing ILS to regulate the execution phase of apoptosis, BIM establishes a foundation for vertical modulation of mitochondria‐dependent intrinsic apoptosis during MIRI.

### 2.6 BIM Vertically Modulates the cGAS–STING Pathway

**FIGURE 6:**
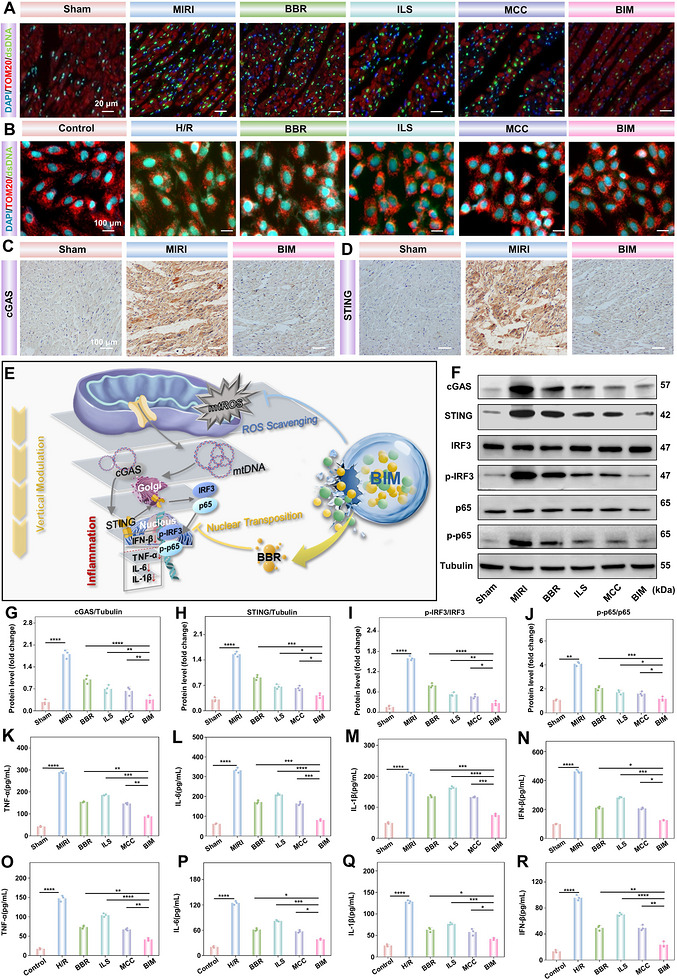
BIM vertically modulates the cGAS–STING pathway. (A) Representative images of myocardial tissue dsDNA (mtDNA marker, green) and TOM20 (red) immunostaining from rats in different groups, scale bar: 20 µm. (B) Representative images of H9c2 cells, dsDNA, and TOM20 immunostaining of different treatment groups; scale bar: 100 µm. (C,D) Representative images of immunohistochemical staining of cGAS and STING in different groups of cardiac tissues; scale bar: 100 µm. (E) Schematic representation of BIM vertically modulates the cGAS–STING pathway. (F–J) Representative images and quantitative analysis of WB of cGAS–STING pathway‐related proteins, including cGAS, STING, p‐p65/p65 and p‐IRF3/IRF3 in different groups of cardiac tissues. (K–N) Detection of TNF‐α, IL‐6, IL‐1β, and IFN‐β in heart tissues of different groups. (O–R) Detection of TNF‐α, IL‐6, IL‐1β, and IFN‐β in H9c2 cells of different groups. Data were expressed as mean ± SE. Statistical significance was analyzed by one‐way ANOVA and Tukey post hoc test. (*n* = 3, ^*^ *p* < 0.05, ^**^ *p* < 0.01, ^***^ *p* < 0.001, ^***^ *p* < 0.0001).

Oxidative stress induced by MIRI exacerbates mitochondrial dysfunction and promotes the leakage of mtDNA into the cytosol, thereby triggering activation of the cGAS–STING signaling pathway [15]. Upon activation, STING translocates from the endoplasmic reticulum to the Golgi apparatus and subsequently drives phosphorylation and nuclear translocation of IRF3 and NF‐κB (p65). This process induces sustained expression of IFN‐β as well as pro‐inflammatory cytokines such as TNF‐α, IL‐6, and IL‐1β, amplifying inflammatory cascades and aggravating myocardial injury [16]. BIM attenuates this process through a dual mechanism. The antioxidant activity of MCC suppresses upstream mtDNA damage and leakage, while the controlled release of BBR directly inhibits phosphorylation and nuclear translocation of IRF3 and p65. Through this coordinated strategy, BIM vertically modulates both the upstream initiating events and downstream effector programs of the inflammatory signaling cascade, thereby effectively alleviating MIRI‐associated inflammatory amplification. Suppression of aberrant activation of the mtDNA–cGAS–STING–IRF3/NF‐κB axis therefore represents a potential mechanism by which BIM mitigates inflammatory injury in MIRI (Figure 6E).

To substantiate the proposed mechanism, mtDNA leakage in myocardial tissue from different groups was first examined using dsDNA and Tom20 co‐staining. As shown in Figure 6A, severe disruption of mitochondrial membrane integrity in the MIRI group resulted in extensive release of mtDNA from damaged mitochondria into the cytosol and even into the extracellular space. In contrast, BIM markedly reduced mtDNA leakage, with an effect that was stronger than that observed with equivalent doses of BBR, ILS, or MCC. Consistent results were also observed at the cellular level, showing close agreement between in vivo and in vitro findings (Figure 6B). These observations directly demonstrate that BIM effectively suppresses mtDNA release. Immunohistochemical analysis further revealed that the numbers of cGAS–STING positive signals in myocardial tissue were significantly increased in the MIRI group, showing 2.1‐ and 2.2‐fold increases compared with the Sham group, respectively. BIM treatment substantially reduced the abundance of cGAS–STING positive signals in MIRI hearts (Figure 6C,D and Figure S14). WB analysis was then performed to assess protein expression within the cGAS–STING signaling pathway in myocardial tissue. Compared with the Sham group, the MIRI group showed marked upregulation of cGAS–STING, p‐IRF3/IRF3, and p‐p65/p65, with increases of approximately 6.5‐, 5.1‐, 10.4‐, and 3.8‐fold, respectively. Treatment with BBR, ILS, MCC, or BIM significantly attenuated these elevations, with BIM exerting the most pronounced inhibitory effect (Figures 6F–J). Collectively, these results indicate that BIM effectively blocks activation of the cGAS–STING signaling pathway during MIRI. At the cellular level, WB analyses showed trends consistent with the in vivo findings. Proteins associated with the cGAS–STING pathway were markedly upregulated in the H/R group, whereas among the four treatments, BIM again exerted the most pronounced inhibitory effect (Figure S15). Inflammatory cytokine levels were then quantified in both in vivo and in vitro models using enzyme‐linked immunosorbent assays (ELISA). In myocardial tissue, compared with the Sham group, levels of the pro‐inflammatory mediators TNF‐α, IL‐6, IL‐1β, and IFN‐β in the MIRI group were significantly elevated to 5.4‐, 6.1‐, 4.8‐, and 6.9‐fold of Sham levels, respectively. Treatment with BBR, ILS, MCC, or BIM at equivalent doses significantly reduced these cytokine levels, with BIM showing the strongest suppression (Figures 6K–N). In vitro, H/R injury similarly induced robust inflammatory responses. Relative to the Control group, TNF‐α, IL‐6, IL‐1β, and IFN‐β levels increased by 8.5‐, 6.1‐, 4.8‐, and 7.2‐fold, respectively. All four treatments significantly attenuated cytokine production under H/R conditions, with BIM again demonstrating the greatest efficacy and clearly outperforming the other agents. Notably, the anti‐inflammatory effect of BBR was substantially stronger than that of ILS, indicating that within the BIM composite system, BBR primarily contributes to the suppression of pro‐inflammatory cytokine production (Figure 6O–R). In summary, BIM preserves mitochondrial membrane integrity through the intrinsic antioxidant activity of its carrier, effectively limits mtDNA leakage that initiates inflammatory signaling, and suppresses terminal pro‐inflammatory cytokine production through controlled release of BBR. Through this coordinated action, BIM vertically modulates the mtDNA–cGAS–STING–IRF3/NF‐κB inflammatory axis and initiates a top‐down intervention to disrupt the vicious cycle between oxidative stress and inflammation during MIRI.

### 2.7 Biocompatibility of BIM

**FIGURE 7:**
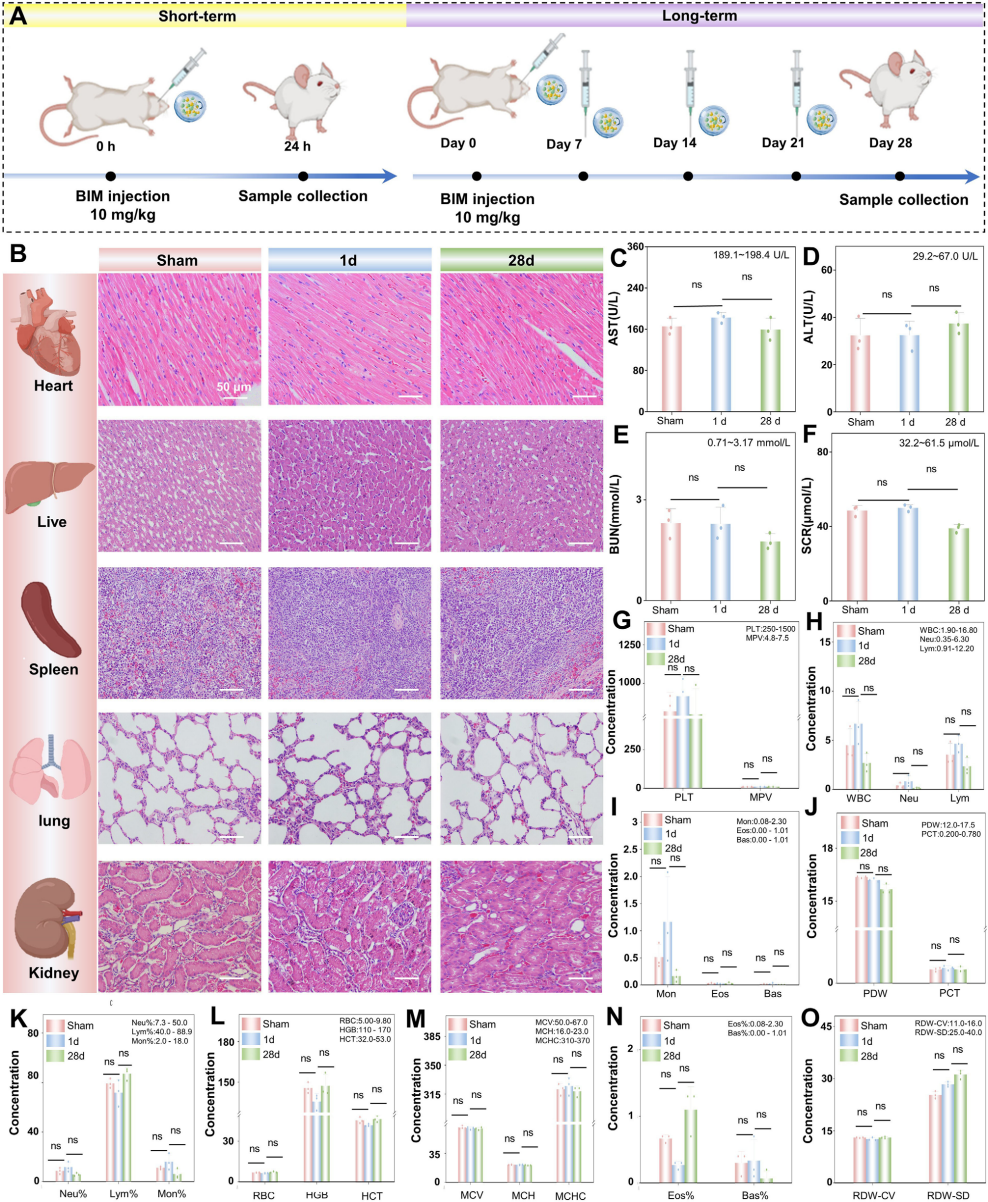
Biocompatibility of BIM. (A) Schematic diagram of short‐term (1 day) or long‐term (28 days) biosafety experiments. (B) HE staining of heart, liver, spleen, lungs, and kidneys 1 or 28 days after BIM injection; scale bar: 50 µm. (C,D) Liver function indexes after 1 or 28 days of administration. (E,F) Renal function after 1 or 28 days of administration. ALT: alanine aminotransferase (normal reference range: 29.2–67.0 U/L); AST: aspartate aminotransferase (normal reference range: 189.1–198.4 U/L); BUN: urea nitrogen (normal reference range: 0.7–3.2 mmol/L); SCR: serum creatinine (normal reference range: 32.2–61.5 mmol/L). (G–O) Values of hematological parameters after 1 or 28 days of BIM administration. WBC: white blood cell count (range: 1.9–16.8); Neu: neutrophil count (range: 0.4–6.3); Lym: lymphocyte count (range: 0.9–12.2); Mon: monocyte count (range: 0.1–2.3); Eos: acidophilic granulocyte count (range: 0.0–1.0); Bas: basophilic granulocyte count (range: 0.0–1.0); Neu%: neutrophil percentage (range: 7.3–50.0); Lym%: lymphocyte percentage (range: 40.0–88.9); Mon%: monocyte percentage (range: 2.0–18.0); Eos%: percentage of eosinophils (range: 0.1–2.3); Bas%: percentage of basophils (range: 0.0–1.0); RBC: number of erythrocytes (range: 5.0–9.8); HGB: hemoglobin (range: 110–170); HCT: erythrocyte cumulative pressure (range: 32.0–53.0); MCV: mean corpuscular volume of erythrocytes (range: 50.0–67.0); MCH: mean corpuscular hemoglobin content of erythrocytes (range: 16.0–23.0); MCHC: mean corpuscular hemoglobin concentration of erythrocytes (range: 310–370); RDW‐CV: coefficient of variation for red cell distribution width (range: 11.0–16.0); RDW‐SD: standard deviation of erythrocyte distribution width (range: 25.0–40.0); PLT: platelet count (range: 250–1500); MPV: mean platelet volume (range: 4.8–7.5); PDW: platelet distribution width (range: 12.0–17.5); PCT: platelet corpuscle (range: 0.2–0.8). There was no significant difference between the Sham and BIM groups. Data are expressed as mean ± SE. Statistical significance was analyzed by one‐way ANOVA using the Tukey post hoc test. (*n* = 3, ns: *p* > 0.05).

Finally, the biocompatibility of BIM was comprehensively evaluated in both in vitro and in vivo settings. The effects of BIM on H9c2 cell viability were first assessed. Cells were exposed to BBR, ILS, MCC, or BIM over a concentration range of 0–160 µg/mL. CCK‐8 assays showed that BIM and MCC exerted minimal effects on cell viability across this entire concentration range. In contrast, BBR and ILS alone induced a clear decline in cell viability at concentrations between 40 and 160 µg/mL (Figure S16). As MCC is primarily composed of PDA, often referred to as artificial melanin, it markedly improves the biosafety profile of the loaded drugs. The in vivo biosafety of BIM was then further examined. As illustrated in Figure 7A, healthy rats were intravenously administered a double therapeutic dose of BIM (10 mg/kg). Tissues were collected 24 h after a single injection for short‐term toxicity assessment, and after repeated administration with sampling on day 28 for long‐term toxicity evaluation. Histological examination revealed no obvious structural damage in major organs, including the brain, heart, liver, spleen, lung, and kidney, in either the short‐term or long‐term treatment groups compared with the Sham group (Figure 7B). Serum biochemical analyses showed that liver function indicators, including alanine aminotransferase (ALT) and aspartate aminotransferase (AST), as well as renal function markers, including serum creatinine (SCR) and blood urea nitrogen (BUN), remained within normal ranges (Figure 7C–F). In addition, complete blood count parameters were all within reference intervals and did not differ significantly from those of the Sham group (Figure 7G–O). Collectively, these results demonstrate that the BIM composite system markedly reduces the toxic side effects associated with free BBR and ILS and exhibits a favorable biosafety profile.

## 3 Discussion

The treatment of MIRI has long been constrained by substantial challenges. Its pathological progression is highly complex and dynamically evolving, involving tightly coupled processes such as mitochondrial dysfunction, oxidative stress, inflammatory amplification, and apoptotic cascades [17, 18]. Interventions targeting a single molecule or relying on a single mode of action are therefore often insufficient to halt disease progression at the system level, and frequently fail to balance efficacy with safety. Accordingly, achieving multi‐level, coordinated, and controllable intervention at critical pathological nodes remains a major barrier to the translational treatment of MIRI. In response to these challenges, this study proposes and validates a microenvironment‐responsive nanomedicine, BIM, designed around the core concept of vertically regulating the pathological network. Rather than simply combining anti‐inflammatory or anti‐apoptotic effects, this strategy implements ordered interventions across different layers of the pathological cascade, enabling integrated control from initiating mechanisms to downstream execution processes. Within this system, the MCC serves as a critical structural and functional backbone. Its favorable biocompatibility and in vivo stability ensure the safe operation of the delivery platform, while its high drug‐loading capacity markedly improves the stability and circulation time of BBR and ILS. Importantly, MCC confers mitochondrial targeting capability, allowing BIM to preferentially accumulate in the most severely damaged mitochondria during MIRI. Furthermore, the early protective effect may partially originate from the intrinsic ROS‐scavenging capability of MCC, which can rapidly neutralize mtROS prior to extensive drug release. By intervening at this pathological core hub, BIM achieves precise and efficient therapeutic modulation while minimizing off‐target exposure and associated risks. Unlike classical mitochondrial‐targeting strategies that rely on lipophilic cations such as triphenylphosphonium (TPP), the mitochondrial localization of MCC is more likely mediated by a non‐classical, affinity‐driven mechanism. Polydopamine (PDA) possesses abundant catechol and amine functional groups, which enable strong interfacial interactions with biomolecules, particularly proteins, via hydrogen bonding, *π*–*π* stacking, and covalent/non‐covalent binding. Previous studies have demonstrated that PDA nanomaterials exhibit broad protein adsorption capacity and can interact with subcellular components, including mitochondrial‐associated proteins [19]. In this study, our mitochondrial isolation and pull‐down experiments further confirm that PDA exhibits binding affinity toward mitochondrial outer membrane proteins. Therefore, the observed mitochondrial localization of MCC may arise from its physicochemical affinity to mitochondrial components rather than ligand‐mediated targeting, representing a non‐canonical but effective mitochondrial interaction mode.

At the mechanistic level, BIM achieves vertical modulation of inflammatory and apoptotic pathways in MIRI through coordinated integration of the complementary functions of BBR and ILS. In this study, the concept of “vertical modulation” refers to a multi‐level intervention of mitochondrial pathological networks, in which BIM acts simultaneously at distinct but functionally connected nodes. Specifically, the melanin shell scavenges mitochondrial ROS, BBR suppresses downstream inflammatory signaling, and ILS inhibits BH3‐only protein‐mediated Bax activation, preventing mitochondrial apoptosis initiation. This framework highlights the advantage of targeting multiple mechanistic layers within the same organelle to achieve comprehensive cardioprotection. As a well‐established anti‐inflammatory agent, BBR has been widely reported to inhibit phosphorylation and nuclear translocation of IRF3 and NF‐κB (p65), thereby reducing the expression of pro‐inflammatory mediators such as IFN‐β, TNF‐α, IL‐6, and IL‐1β [20]. In this study, we further demonstrate that under MIRI‐associated oxidative stress, aberrant activation of the mtDNA‐driven cGAS–STING pathway represents a key driver of inflammatory amplification. BIM effectively suppresses sustained activation of the cGAS–STING axis, enabling inhibition at both the initiation and transcriptional execution levels of inflammatory signaling and revealing a deeper mechanistic basis for its anti‐inflammatory effects. In parallel, ILS primarily exerts anti‐apoptotic activity by inhibiting BH3‐only protein–mediated Bax activation and MOMP, thereby blocking Cyt C release and the caspase‐3–dependent apoptotic cascade. Importantly, BBR and ILS do not act independently when delivered by the MCC carrier. Instead, they converge on mitochondria as a shared pathological hub, enabling coordinated regulation of both inflammatory and apoptotic axes. This same‐target, multi‐level regulatory paradigm fundamentally distinguishes BIM from conventional combination therapies and underpins its enhanced therapeutic efficacy.

Notably, this study evaluated the potential advantages of BIM from the perspective of overall biosafety. Previous reports have indicated that BBR may carry a risk of systemic toxicity at higher doses or under certain administration conditions, which can limit the safety window of its standalone use [21]. In the present study, even under relatively high dosing conditions, animals treated with BIM showed preserved structural integrity of major organs, including the heart, liver, spleen, lung, and kidney, with no evident pathological abnormalities. In parallel, liver and renal function indices as well as complete blood count parameters remained within normal physiological ranges, indicating favorable biocompatibility of BIM. Compared with the potential adverse effects of free‐nature compounds at high doses, the improved biosafety profile observed for BIM suggests that its delivery strategy effectively broadens the therapeutic safety window. This advantage is likely attributable to the ischemic microenvironment–responsive release behavior of MCC, which reduces transient systemic exposure to the active compounds in circulation, thereby enhancing overall safety while maintaining therapeutic efficacy. Collectively, these findings further underscore the critical role of rational delivery systems in optimizing the balance between efficacy and safety. Overall, this work not only presents a mitochondria‐targeted combination nanotherapeutic for MIRI, but more importantly, validates the feasibility of a treatment paradigm based on vertical modulation of pathological networks. Although the present study demonstrates that BIM effectively protects mitochondria and attenuates cardiomyocyte apoptosis in early‐stage myocardial ischemia‐reperfusion injury, several limitations should be acknowledged. First, while ILS was shown to inhibit BH3‐only protein‐mediated Bax activation, the precise molecular interactions and downstream consequences require further mechanistic investigation to fully elucidate its anti‐apoptotic action. Second, although BBR and ILS were co‐delivered and observed to act synergistically, the extent and mechanistic basis of this synergy remain to be clarified. Third, while the protein pull‐down assay indicated that MCC could interact with mitochondrial outer membrane‐associated proteins such as TOM and VDAC, this finding mainly suggests the protein‐adsorption capability of the PDA‐based surface and does not fully prove that BIM actively or selectively binds to mitochondria in vivo. Therefore, the mitochondrial enrichment of BIM may result from the combined effects of particle size, surface charge, PDA–protein affinity, and mitochondrial membrane or electrochemical alterations under oxidative stress. Fourth, although the early therapeutic effect of BIM may be largely attributed to the ROS‐scavenging capacity of MCC, the later anti‐inflammatory and anti‐apoptotic effects are likely associated with sustained drug release. Thus, future studies should further distinguish the temporal contributions of MCC‐mediated ROS clearance and drug‐release‐mediated therapeutic effects using longer‐term release profiles, such as 6 and 12 h. Finally, while ex vivo fluorescence tracing and electron microscopy confirmed myocardial targeting and mitochondrial localization of BIM, in vivo imaging experiments would provide more direct evidence of biodistribution and dynamic accumulation in infarcted myocardium. Future studies should focus on dissecting the detailed ILS–Bax interaction network, quantitatively assessing the synergistic effects between BBR and ILS, clarifying the temporal relationship between ROS scavenging and drug release, and incorporating longitudinal in vivo imaging to monitor BIM pharmacokinetics and organ‐specific targeting. Addressing these points will provide a more comprehensive understanding of the therapeutic potential of BIM and strengthen its translational relevance.

## 4 Conclusion

In summary, this study proposes and validates a therapeutic strategy that targets mitochondria as a central pathological hub and alleviates MIRI through vertical modulation of interconnected networks involving oxidative stress, inflammation, and apoptosis. The mitochondria‐targeted nanotherapeutic BIM developed here leverages the antioxidant properties and damaged‐mitochondria enrichment capacity of MCC to enable coordinated delivery and functional complementarity of the anti‐inflammatory agent BBR and the anti‐apoptotic agent ILS at critical pathological nodes. Mechanistically, BIM scavenges mtROS, stabilizes mitochondrial function, and suppresses aberrant activation of the mtDNA‐associated cGAS–STING signaling pathway, thereby jointly attenuating inflammatory amplification and mitochondria‐dependent apoptosis. In a rat model of MIRI, BIM markedly reduced myocardial injury at a low dose while exhibiting a favorable biosafety profile. Together, these findings establish a feasible therapeutic paradigm based on mitochondria‐centered, vertical modulation of pathological networks and provide new conceptual and practical insights for the treatment of MIRI and related cardiovascular diseases.

## Conflicts of Interest

The authors declare no conflicts of interest.

## Supporting information




**Supporting File**: advs76519‐sup‐0001‐SuppMat.docx.

## Data Availability

The data that support the findings of this study are available from the corresponding author upon reasonable request.
