# Supplementary material for: Microenvironment‐Responsive Nanomedicine Enables Vertical Modulation of Mitochondrial Pathological Networks in Myocardial Ischemia–Reperfusion Injury
Source: Adv Sci (Weinh). 2026 Jul 13:e76519. Online ahead of print. doi: 10.1002/advs.76519 (PMC13360117; doi:10.1002/advs.76519)
Supplement: Supplementary file 1 — Supporting File: advs76519‐sup‐0001‐SuppMat.docx. [file ADVS-9999-e76519-s001.docx]

**Microenvironment-responsive nanomedicine enables vertical modulation of mitochondrial pathological networks in myocardial ischemia–reperfusion injury**

*Jue Wang^1,2^*^†^*, Jia Zhou^1,6,7^*^†^*, Wenqin Yuan^2^, Tianjiao Zhao^2^, Yunying Huang^2^, Yingci Xia^2^,*

*Yuting Lin^2^, Fei Li^2^, Qiong Huang^1,3*^, Chong Liu^2*^, Kelong Ai^2,4,5*^, Qun Qin^1,3,6*^*

^1^Department of Pharmacy, Xiangya Hospital, Central South University, Changsha, 410008, China.

^2^Xiangya School of Pharmaceutical Sciences, Central South University, Changsha, 410013, China.

^3^National Clinical Research Center for Geriatric Disorders, Xiangya Hospital, Central South University, Changsha, 410008, China.

^4^Hunan Provincial Key Laboratory of Cardiovascular Research, Xiangya School of Pharmaceutical Sciences, Central South University, Changsha, 410013, China.

^5^Key Laboratory of Aging-related Bone and Joint Diseases Prevention and Treatment, Ministry of Education, Xiangya Hospital, Central South University, Changsha, 410008, China.

^6^Hunan Province International Science and Technology Innovation Cooperation Base for Early Clinical Trials of Biological Agents, Xiangya Hospital, Central South University, Changsha, 410008, China.

^7^Department of Pharmacy, Yizhang County People's Hospital, Chenzhou, 424299, Hunan, China.

**^†^ The authors Jue Wang and Jia Zhou contributed equally to the work.**

***Corresponding authors**: qionghuang@csu.edu.cn (Prof. Qiong Huang), llchong@csu.edu.cn (Prof. Chong Liu), aikelong@csu.edu.cn (Prof. Kelong Ai), 2395038683@qq.com (Prof. Qun Qin).

**Supporting Information**

**Methods and Materials**

The main materials are presented in the **Supplementary Table 1** below.

**Supplementary Table 1: Main materials**

| Reagents | Source | Catalogue |
| --- | --- | --- |
| Uniform polystyrene microspheres | Macklin | C11828028 |
| Dopamine hydrochloride | Aladdin | L2010199 |
| Xylene | Aladdin | G2115262 |
| Tetrahydrofuran | Macklin | C15090247 |
| Tris | Biofroxx | Biofroxx |
| Sodium dodecyl sulfate | Aladdin | I2114329 |
| Methionine | Macklin | L812760 |
| Riboflavin | Macklin | R817215 |
| Nitro-blue tetrazolium | Macklin | N814596 |
| Terephthalic acid | Macklin | D835927 |
| Hydrogen peroxide | Sigma | 216763 |
| Ferrous sulfate | Aladdin | F116338 |
| Triphenyl tetrazolium chloride | Sigma | T8877 |
| Reactive oxygen species assay kit | Solarbio | CA1410 |
| Mitochondrial membrane potential assay kit with JC-1 | Beyotime | C2006 |
| Mitosox Red Mitochondrial Superoxide Indicator | Yeasen | 40778ES50 |
| Cell Counting Kit-8 | Beyotime | C0038 |
| Malondialdehyde (MDA) Test Kit | Jiancheng Bioengineering Institute | A003-1 |
| MitoTracker® Deep Red FM | Yeasen | 40743ES50 |
| ER-Tracker Red | Beyotime | C1041 |
| Golgi-Tracker Red | Beyotime | C1043 |
| Hoechst 33342 | Beyotime | C1022 |
| TUNEL Apoptosis Detection Kit (FITC) | Yeasen | 40306ES50 |
| Enhanced BCA Protein Assay Kit | Beyotime | P0010 |
| Antifade Mounting Medium with DAPI | Beyotime | P0131 |
| Enhanced ATP Assay Kit | Beyotime | S0027 |
| Annexin V-FITC/PI Apoptosis Detection Kit | Yeasen | 40302ES50 |
| CK Assay Kit | Jiancheng Bioengineering Institute | A032-1-1 |
| Cell Mitochondria Isolation Kit | Beyotime | C3601 |
| Tissue Mitochondria Isolation Kit | Beyotime | C3606 |

**Synthesis of BIM**

PDA was synthesized in situ on the surface of PS with a diameter of 300 nm, and the PS were expanded with emulsifier to form a single opening. After removing the PS, MCC are formed. Subsequently, polyethylene glycol (PEG) was attached to the MCC surface to improve its biocompatibility and BIM was synthesized by loading BBR and ILS. Specifically, 36 mg of Tris was added to 25 mL of ultra-pure water in a flask and dissolved by sound waves. Polystyrene nanospheres and DA were successively added to the flask and stirred at 25℃ for 12 h. The precipitate is washed three times in ultra-pure water and preserved. Directly add "Tris 36 mg + ultra-pure water 25 mL" + "centrifugal precipitate + ultra-pure water 3 mL" + " DA 30 mg + ultra-pure water 3 mL" and stir at 25℃. After 12 h, wash three times with ultra-pure water and disperse in 2.5 mL ultra-pure water. 0.3 g sodium dodecyl sulfate was added to 28 mL ultra-pure water and 0.3 mL xylene and emulsified by ultrasonic vibration. The emulsifier and 2.5 mL nanospheres were stirred at room temperature for 6 h, and then 60 mL ethanol was added and stirred for 5 minutes. After the reaction was stopped, the supernatant was centrifuged and washed three times with ethanol. Then add 30 mL tetrahydrofuran, stir for 4 h, centrifuge and discard the supernatant, wash tetrahydrofuran three times, ethanol three times, ultra-pure water three times to obtain MCC. PEG is attached to the surface of MCC. Add "Tris 36 mg + NH2-PEG 0.1g + ultra-pure water 5 mL" + "MCC precipitate + ultra-pure water 25 mL" directly into the flask and stir at 25℃. After 12 h, wash three times with ultra-pure water and disperse in 10 mL ultra-pure water. In addition, BBR and ILS were loaded into the MCC prepared above: 20 mg BBR and 20 mg ILS were dissolved in 1mL dimethyl sulfoxide, respectively. Add 1 mL BBR, 1 mL ILS and 3 mL MCC to the flask and stir at 25℃ for 24 h. The sediment is then washed three times with ultra-pure water. BIM is obtained by removing supernatant.

**Synthesis of BIM-FITC**

BIM (10 mg) and NH_2_-PEG-NH_2_ (12.5 mg) were dissolved in 10 mL Tris solution with pH 8.5 and stirred at 25℃ for 12 h. It was then washed three times with ultra-pure water to precipitate and dispersed in 8 mL ultra-pure water. Dissolve 2 mg FITC in 2 mL Dimethyl sulfoxide. BIM-FITC was obtained by pouring the two solutions into a round-bottomed flask, stirring at room temperature for 6 h, and dialysis for 24 h to remove the unreacted FITC.

**The drug loading capacity of MCC**

The standard concentration curves of BBR and ILS were prepared by ultraviolet spectrophotometer to determine the drug loading of MCC. The supernatant was 100 µL and the absorbance was measured by ultraviolet spectrophotometer. The concentrations of BBR and ILS in the supernatant were calculated according to the standard curve. The calculation formula of drug loading efficiency is "(total mass of (BBR+ ILS) - concentration of supernant (BBR+ ILS) × reaction volume)/total mass of MCC × 100%".

**The drug release of BIM**

In brief, BIM containing a total mass of 40 mg of BBR (Berberine) and ILS (Isoliensinine) were dispersed in PBS solution (pH 6.0 or pH 7.4) and then incubated in a thermostatic water bath under gentle stirring (37 °C). At predetermined time points (0.25, 0.5, 1, 2, 3 h), the sample solutions were centrifuged at 12000 rpm for 5 min. The supernatants were collected, and the absorbance of ILS and BBR was measured at 280 nm and 345 nm, respectively, using a UV spectrophotometer. The cumulative release of ILS and BBR was periodically recorded according to the standard concentration curves.

**The characterization of BIM and MCC**

The appearance and morphology of MCC and BIM were observed by TEM images taken with TECNAI G2 high-resolution transmission electron microscope. Zeta potentials of MCC and BIM were determined by Zetasizer Nano ZS90.The elemental states of MCC and BIM were analyzed by XPS measuring instrument (VG ESCALAB MKII).

**Superoxide anion scavenging assay *in vitro***

The ability of MCC and BIM to remove O_2_·^-^ was determined by nitroblue tetrazole (NBT) method. Simply put, different concentrations of MCC or BIM (0，1, 2, 4, 8, 16 μg/mL) are mixed with methionine (0.1 M), riboflavin (20 μM), NBT (0.01 M), PBS (0.1 M, pH 7.4), and ultrapure water. Then, it was exposed to ultraviolet light for 8 minutes. Finally, the absorbance was measured at 560 nm to analyze the ability of MCC or BIM to clear O_2_·^-^.

**Scavenging hydroxyl radicals**

The Fenton reaction between FeSO_4_ and H_2_O_2_ catalyzes the formation of ·OH. Terephthalic acid (TA) can react with ·OH to produce fluorescent substances, which can be used as an indicator of the ·OH content in solution. Briefly, different concentrations of MCC or BIM (0, 2, 4, 8, 16 μg/mL) were mixed with disodium terephthalate (0.1 mM), ferrous sulfate (0.05 mM), H_2_O_2_ (10 mM), PBS (0.01 M, pH 7.4), and ultrapure water. After reacting in the dark for 6 min, the mixture was transferred to a test tube and scanned for the corresponding fluorescence intensity at the excitation wavelength of 320 nm.

**H_2_O_2_ scavenging assay *in vitro***

The ability of MCC and BIM to scavenge H_2_O_2_ was determined by UV spectrophotometry. MCC or BIM (50 µg/mL) were mixed with different concentrations of H_2_O_2_ (0, 1, 2, 4, 8, 16 μM), and incubated in the dark for 12 h. The scavenging rate of H_2_O_2_ was determined by UV absorption.

**ONOO^-^ scavenging assay *in vitro***

The ability of MCCs and BIM to scavenge ONOO^-^ was determined by UV spectrophotometry. MCC or BIM (50 µg/mL) were mixed with different concentrations of ONOO^-^ (0, 1, 2, 4, 8, 16 μM), and the reaction was incubated in the dark for 12 h. Then the ONOO^-^ was determined by detecting its UV absorption.

**Mitochondrial colocalization assay**

The subcellular localization of BIM was observed by fluorescence microscopy. Briefly, 2 × 10^4^ H9C2 cells were inoculated in 24-well plates, treated with BIM-FITC for 24 h, and then washed with PBS for three. The cells were then stained with Mito-tracker (Yeasen, 40743ES50) and Hoechst 33342 (Beyotime, C1022) according to the instructions. Images were taken with a fluorescence microscope and co-localization values were calculated using ImageJ software.

**MIRI model**

SD rats (male, 6-8 weeks, 200-220 g) were purchased from Hunan STA Laboratory Animal Co. Ltd (SYXK (XIANG) 2020-0019) (Changsha, China). All animals were housed under standard conditions (24 ± 2 °C, 12 h light/dark cycle) in a specific pathogen-free facility and acclimatized for one week with standard chow and water provided ad libitum. All animal experiments were approved by the Animal Care and Use Committee of Central South University (Approval No. XMSB-2023-0066). The anterior descending branch of the left coronary artery was ligated and relaxed to establish the rat MIRI model. After anesthetizing SD rats with sodium pentobarbital, the skin was incised along the left chest at the level of the 3rd ~ 4th intercostal space. The chest muscles were directly separated with forceps to expose the heart. Then the anterior descending branch of the left coronary artery was ligated with 5-0 silk thread. After 1 h of ischemia, the suture was removed for recanalization of the anterior descending branch of the left coronary artery. Drugs were administered via sublingual vein 5 minutes before reperfusion, and specimens were taken 3 h after reperfusion. The animals used for echocardiographic assessment underwent an extended reperfusion period of 24 h prior to measurement. Fluorescence values were calculated using ImageJ software.

***In vivo* fluorescence imaging**

The experimental animals were subjected to MIRI modeling, and then BIM-FITC was injected intravenously for 10 min, 30 min, 1 h, 2 h, and 3 h. Heart, liver, spleen, lungs, kidneys, and brain tissues were taken and immersed in PBS. The fascia and blood vessels on the tissue surface were peeled off and imaged under a Leica fluorescence microscope. Meanwhile, the heart was sectioned and TTC stained. Fluorescence values were calculated using ImageJ software.

**Fluorescence imaging of tissue frozen sections**

After experimental sampling hearts were frozen at -80 °C and then at -20 °C for 30 min. Specimens were embedded in OCT and sectioned into 10 μm sections. The sections were washed three times with PBS for 5 min and then incubated with 0.05% Triton for 5 min. The sections were washed again, sealed with DAPI-Fluoromount-G, and photographed by fluorescence microscopy. Fluorescence values were calculated using ImageJ software.

**Triphenyl Tetrazolium Chloride (TTC) Staining**

After sampling, hearts were frozen at -20 °C for 1 h and then cut into thin sections. The heart sections were incubated with 1% TTC solution for 12 min at 37 °C in the dark, and the sections were turned over to ensure staining on both sides. After staining, the sections were fixed with 4% paraformaldehyde, photographed with a camera, and finally the infarct area was calculated by Image J.

**Determination of serum CK concentration**

For measurement of CK concentration, the blood sample was collected at the end of reperfusion, then the serum was isolated for CK concentration assay according to the instructions (Nanjing Jiancheng Bioengineering Institute, A032-1-1).

**Determination of MDA** **concentration**

MDA concentration was used to assess lipid peroxidation levels. After tissue homogenization, all samples were centrifuged at 12000 × g for 15 minutes at 4 °C. The supernatant was collected for subsequent analysis. MDA concentration was measured according to the kit instructions (Nanjing Jiancheng Bioengineering Institute, A003-1).

**HE staining**

After the experiment, the samples of heart, liver, lung, spleen and kidney were fixed with 4% paraformaldehyde, embedded in paraffin, and cut into 5 μm thick sections. After deparaffinization, the sections were stained with hematoxylin and eosin, respectively. Finally, they were observed under a microscope and photographed.

**Determination of ROS production in myocardial tissue**

For ROS staining of tissues, hearts were excised and immediately prepared as frozen sections. Sections were incubated with DHE working solution and photographed under a fluorescence microscope. Fluorescence values were calculated using ImageJ software.

**Transcriptome Sequencing**

RNA was extracted from cardiac tissue, total RNA was extracted using standard TRIzol protocols, and cDNA libraries were synthesized by mRNA enrichment. Sequencing was performed on a high-throughput sequencing platform, and the resulting data were analyzed for quantitative gene analysis and differential gene expression analysis, as well as enrichment analysis.

**TUNEL staining**

Paraffin sections of heart tissue were prepared. After paraffin sections were deparaffinized, they were stained according to the instructions of the TUNEL kit (Yeasen, 40306ES50), and the nuclei were stained with DAPI. Finally, images were taken using a fluorescence microscope. Positive values were calculated using ImageJ software.

**Immunohistochemistry**

Paraffin sections were dewaxed and hydrated and repaired with EDTA antigen. Incubate by adding appropriate amount of endogenous peroxidase blocker. 5% BSA blocking, add appropriate amount of cGAS and STING antibody, incubate at 4°C overnight. Incubate with appropriate amount of reaction enhancement solution, add appropriate amount of enzyme labeled goat anti-mouse and rabbit IgG polymers and incubate for 30 min, add DAB solution and incubate, terminate the reaction by immersion in tap water. Sections were counterstained with hematoxylin and rinsed with tap water. Sections were differentiated with 1% ethanol hydrochloride and then soaked in tap water to turn them blue. Sections were dehydrated and sealed with neutral resin, observed under a histochemical microscope and photographed. The list of antibodies used for immunohistochemistry is shown in **Supplementary Table 2** below.

**Supplementary Table 2: Antibody list 1**

| Primary/Secondary antibody | Company | Cat. No | Dilution |
| --- | --- | --- | --- |
| cGAS | Proteintech | 29958-1-AP | 1:500 |
| STING | Proteintech | 19851-1-AP | 1:500 |

**Inflammatory factor detection**

ELISA kits (Table 3) were used to assess the levels of IL-1β, IL-6, TNF-α, and IFN-β in cardiac tissue or H9c2 cells. The list of ELISA kits is shown in **Supplementary Table 3** below.

**Supplementary Table 3: List of ELISA kits**

| Primary/Secondary antibody | Company | Cat. No |
| --- | --- | --- |
| Rat TNF-α (Tumor Necrosis Factor Alpha) ELISA Kit | Elabscience | E-EL-R2856 |
| Rat IL-6 (Interleukin 6) ELISA Kit | Elabscience | E-EL-R0015 |
| Rat IL-1β (Interleukin 1 Beta) ELISA Kit | Elabscience | E-EL-R1002 |
| Rat IFN-β (Interferon Beta) ELISA Kit | Elabscience | E-EL-R0545 |

**Immunofluorescence staining**

H9c2 cells were grown on coverslips, washed twice with PBS and fixed with 4% paraformaldehyde. after three washes with PBS, cells were permeabilized by incubation with Triton X-100. Cells were blocked with blocking buffer (2% BSA in PBS) for 1 h at 25°C. Cells were incubated with dsDNA and TOM20 primary antibody overnight at 4°C. Cells were washed with PBS and then exposed to Alexa Fluor-488 and 594-conjugated secondary antibodies for 30 min. Cell nuclei were stained with DAPI solution. Images were obtained using fluorescence confocal microscopy. Tissues were stained for immunofluorescence, paraffin sections were deparaffinized, and the staining procedure was performed as above. The list of antibodies used for immunofluorescence staining is shown in **Supplementary Table 4** below.

**Supplementary Table 4: Antibody list 2**

| Primary/Secondary antibody | Company | Cat. No | Dilution |
| --- | --- | --- | --- |
| Anti-dsDNA antibody | Abcam | AB27156 | 1:500 |
| TOM20 Polyclonal antibody | Proteintech | 11802-1-AP | 1:500 |
| Alexa Fluor™ 555 | Invitrogen | A21428 | 1:500 |
| Alexa Fluor™ 488 | Invitrogen | A11029 | 1:500 |

**Protein preparation and western blot**

Rat heart tissue/H9c2 cells were lysed using lysate on ice for more than 30 min. The lysate was centrifuged at 12000 rpm for 15 min at 4°C, and the protein concentration of the supernatant was analyzed using a BCA kit (Beyotime, China). Each sample (20 μg) was separated by SDS-PAGE gel and transferred to a PVDF membrane. The membranes were soaked in 5% milk in TBST buffer for 1 h at room temperature, and then the primary antibody was incubated with the following antibodies overnight at 4°C. The membranes were washed three times with TBST. The membrane was then washed three times with TBST and then incubated with mouse/rabbit secondary antibody (1:3000) for 1 h at room temperature. Bands were displayed with a gel documentation system (Bio-Rad, USA) and quantified with ImageJ software. The list of antibodies used for western blot is shown in **Supplementary Table 5** below.

**Supplementary Table 5: Antibody list 3**

| Primary/Secondary antibody | Company | Cat. No | Dilution |
| --- | --- | --- | --- |
| Tubulin | Affinity | AD7011 | 1:4000 |
| p-p65 | Affinity | AF2006 | 1:1000 |
| p65 | Affinity | BF8005 | 1:1000 |
| p-IRF3 | Affinity | AF2436 | 1:1000 |
| IRF3 | Affinity | DF6895 | 1:1000 |
| Cyt c | Proteintech | 10993-1-AP | 1:5000 |
| Caspase-3/Cleaved Caspase-3 | Wanleibio | WL02117 | 1:500 |
| Bax | Abcam | ab32503 | 1:5000 |
| Bcl2 | Affinity | BF9103 | 1:1000 |
| cGAS | Proteintech | 29958-1-AP | 1:1000 |
| STING | Proteintech | 19851-1-AP | 1:1000 |
| TOM7 | Affinity | DF12045 | 1:1000 |
| TOM34 | Proteintech | 12196-1-  AP | 1:2000 |
| TOM70 | Cell signaling  technology | 65619 | 1:1000 |
| TOM40 | Cell signaling  technology | 55959 | 1:1000 |
| VDAC 1/2 | Proteintech | 10866-1-  AP | 1:2000 |
| Citrate Synthetase | Immunoway | YM960095 | 1:5000 |
| ATP5A | Immunoway | YM8161 | 1:3000 |
| CPT-1 | Immunoway | YM8449 | 1:3000 |
| Goat anti Rabbit IgG H&L (HRP) | Abclonal | AS014 | 1:6000 |
| Goat anti Mouse IgG H&L (HRP) | Abclonal | AS003 | 1:5000 |

**H9c2 cell culture**

H9c2 cells were purchased from Shanghai Institute of Cell Biology (Shanghai, China). DMEM medium containing 10% fetal bovine serum was used for culture. Cells were cultured at 37°C, 5% CO_2_ and humidified environment for survival.

**Construction of H9c2 cell hypoxia/reoxygenation model**

H9c2 cells were inoculated at a density of 1×10^4^/cm^2^ and then randomly divided. After growth to 70-80%, the cell culture medium of CoCl_2_, BBR, ILS, MCC and BIM groups was replaced with that of 600 μM CoCl_2_. BBR, ILS, MCC and BIM groups contained four drugs respectively, and were cultured for 24 h. The CoCl_2_-containing culture medium was replaced with the normal medium containing the corresponding drugs only. culture for 6 h. At the end of culture, H9c2 cells were collected for subsequent studies.

**H9c2 cell CCK8 assay**

After overnight incubation in 96-well plates (1×10^5^/well), cells were treated with different concentrations of BBR, ILS, MCC and BIM for 24 h. Subsequently, the medium was replaced with culture medium containing 10% CCK8 and incubated at 37°C for 60 min. Absorbance was measured at 450 nm using an enzyme meter.

**Measurement of Mitosox level in H9c2 cells**

H9c2 cells were inoculated in 24-well plates (2×10^4^/well). After the cells were grown to a suitable density, each group was stimulated with BBR, ILS, MCC and BIM for 24 h under CoCl_2_ conditions. Then each group was incubated with whole culture solution containing only their corresponding drugs for another 6 h. The cells were stained according to the instructions (Yeasen, 40778ES50) and finally analyzed by fluorescence microscopy.

**Measurement of ROS levels in H9c2 cells**

Cells were treated as above, then stained according to the instructions of the ROS Detection Kit (Solarbio, CA1410) and finally analyzed by fluorescence microscopy.

**Mitochondrial membrane potential measurement**

Mitochondrial membrane potential was measured using the JC-1 kit (Beyotime, C2006). Cells were processed as described above, stained according to the kit instructions, and analyzed by fluorescence microscopy.

**Detection of ATP generation**

ATP production was assayed using the ATP Assay Kit (Beyotime, S0027). Briefly, cells were treated as described above and assayed according to the kit instructions.

**Analysis of apoptosis level *in vitro***

The Annexin V-FITC Apoptosis Detection Kit (Yeasen, 40302ES50) was used to detect the level of apoptosis in H9c2 cells. Briefly, cells were treated as described above and assayed according to the kit instructions.

***In vivo* biosafety analysis**

SD male rats were randomly divided into two groups. Rats in the experimental test group were given BIM 10 mg/kg; the sham-operated group was given an equal amount of saline. The rats in each group were euthanized 1 and 28 days after the administration of BIM (once every 7 days via sublingual vein). Major organs (heart, liver, spleen, lungs and kidneys) and blood were subsequently collected. Subsequently, pathological changes in each organ were analyzed by HE staining; hematology (Sysmex XE-2100, Kobe, Japan) was analyzed for the following blood parameters: leukocytes, erythrocytes, and hemoglobin, etc.; and liver and kidney functions (ALT, AST, BUN, and CRE levels) were analyzed using multifunctional enzyme markers.

**Molecular docking**

The protein structure of the BH3 domain binding to Bax was downloaded from the PDB database, with its PDB ID as 4BD2. The SMILE structure of ILS was downloaded from the PubChem database. Molecular docking was performed using AUTODOCK VINA software to study the direct interaction between the BH3 domain and Bax, as well as the interaction between the BH3 domain bound to ILS and Bax. The effect of ILS binding on the binding ability between the BH3 domain and Bax was investigated. All results were visualized and analyzed using PyMOL software.

**Synthesis of Fe_3_O_4_@MCC and binding affinity with mitochondrial proteins**

Fe_3_O_4_@MCC was synthesized by coating dopamine on the surface of Fe_3_O_4_ magnetic nanoparticles. In brief, take 1.5 mL of Fe_3_O_4_ magnetic nanoparticles, centrifuge and discard the supernatant. The precipitate was then washed three times with ultrapure water and set aside. “36 mg of Tris +25 mL ultrapure water” + “centrifuged precipitate +2 mL ultrapure water” + “30 mg of dopamine hydrochloride +3 mL ultrapure water” were added directly to the flask and stirred (500 rpm) for 12 h at 25 ℃. The precipitate was then washed three times with ultrapure water. Fe_3_O_4_@MCC was collected by removing the supernatant. Mitochondria isolated from H9c2 cells were lysed to prepare protein extracts, which were then incubated with Fe_3_O_4_@MCC for 6 h at 4 ℃. After that, the Fe_3_O_4_@MCC -protein complex was magnetically separated and washed three times. After trypsin digestion, captured mitochondrial proteins were analyzed by WB.

**Echocardiography**

After anesthesia with isoflurane, rats were positioned on a heated platform. Echocardiographic assessments of left ventricular function using a Vevo2100 Imaging System were conducted at 24 hours post-surgeries. Parameters including EDV, ESV, LVIDd and LVIDs were determined from M-Mode echocardiography and All data analyses were conducted by a blinded investigator to treatment groups. EDV and ESV represented left ventricular end-diastolic volume and left ventricular end-systolic volume. LVEF was derived as LVEF (%) ＝ (EDV - ESV)/EDV × 100%. LVIDd and LVIDs represented ventricular diameters at diastole and systole ends, respectively. LVFS was derived as LVFS (%) = (LVIDd - LVIDs)/LVIDd × 100%.

**Statistical analyses**

All experiments were performed in parallel at least 3 times and results are expressed as mean ± standard error. Data were analyzed using GraphPad Prism, Image J, and Origin. Statistics were analyzed by one-way ANOVA, and differences were considered statistically significant at *P* < 0.05.

**Experimental data**


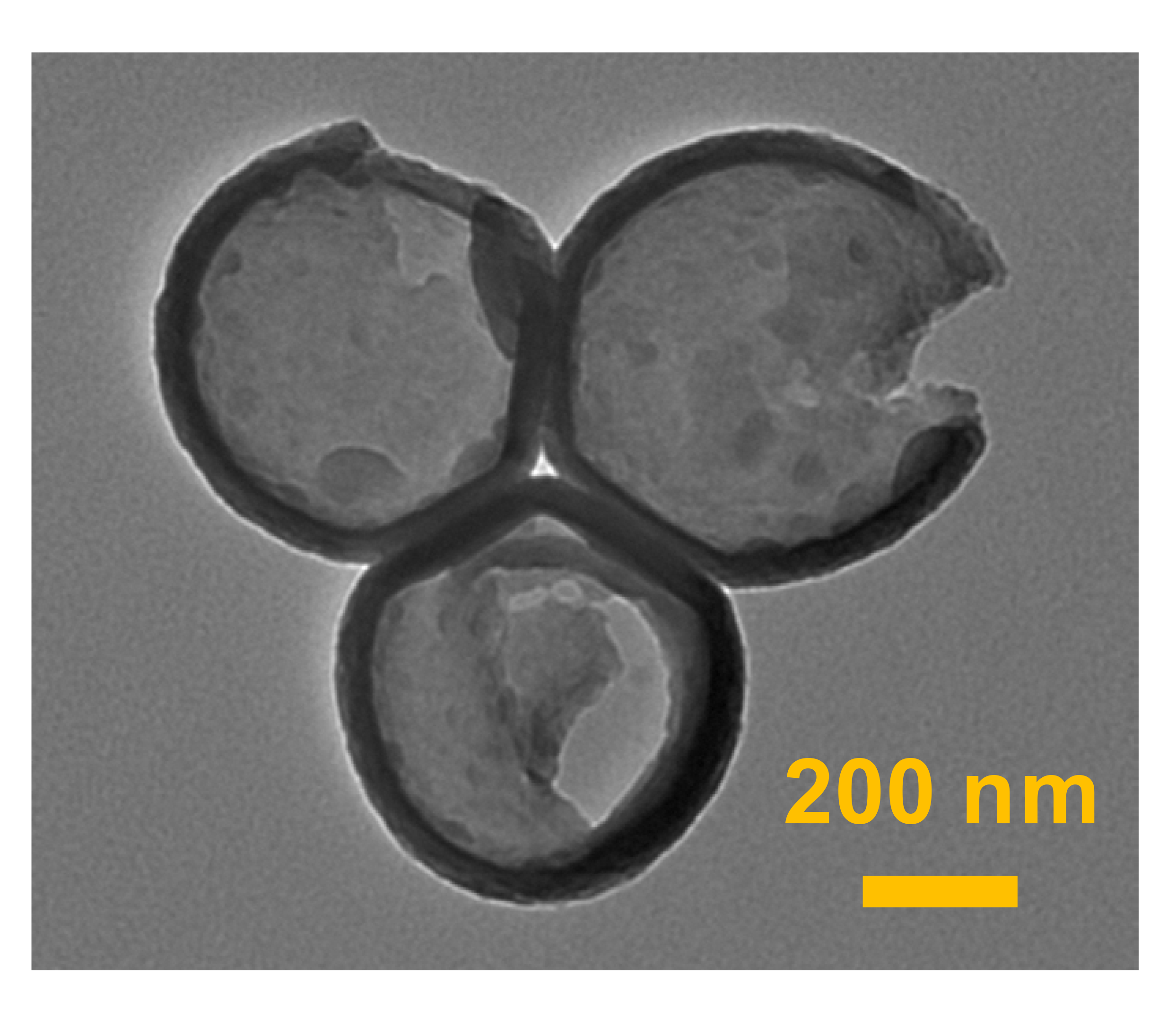


**Figure S1. TEM image of MCC, scale bar: 200 nm.**

**
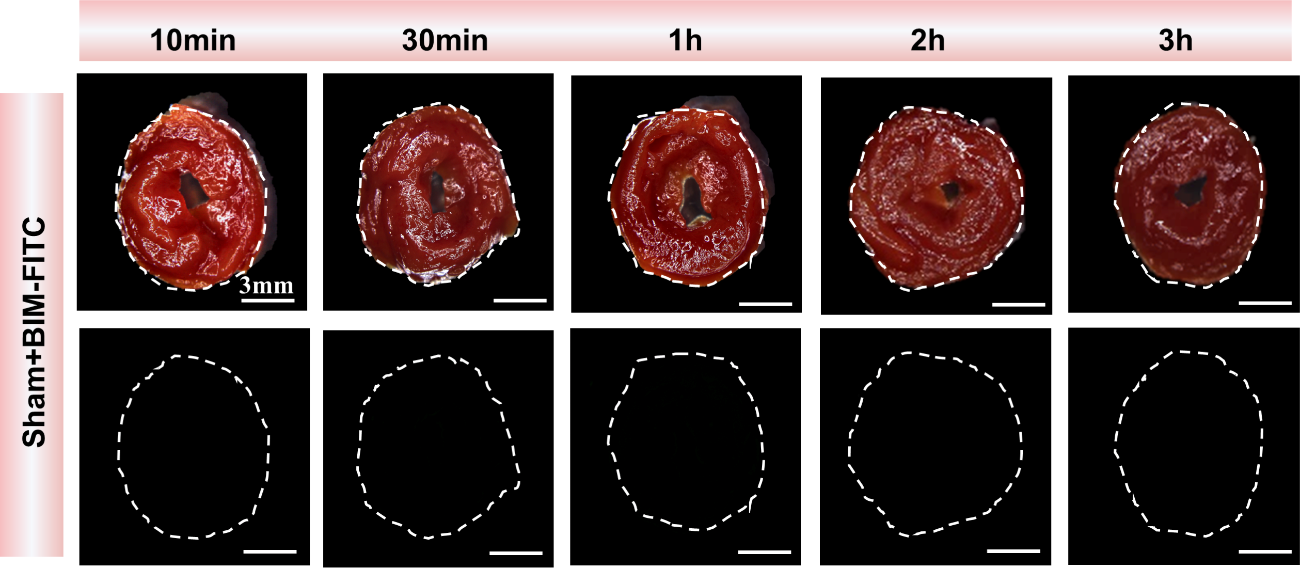
**

**Figure S2. Representative images of Sham-FITC fluorescence imaging and bright field images of the heart at 0 min, 10 min, 30 min, 1h, 2h, and 3h after MIRI rats, scale bar: 3**

**
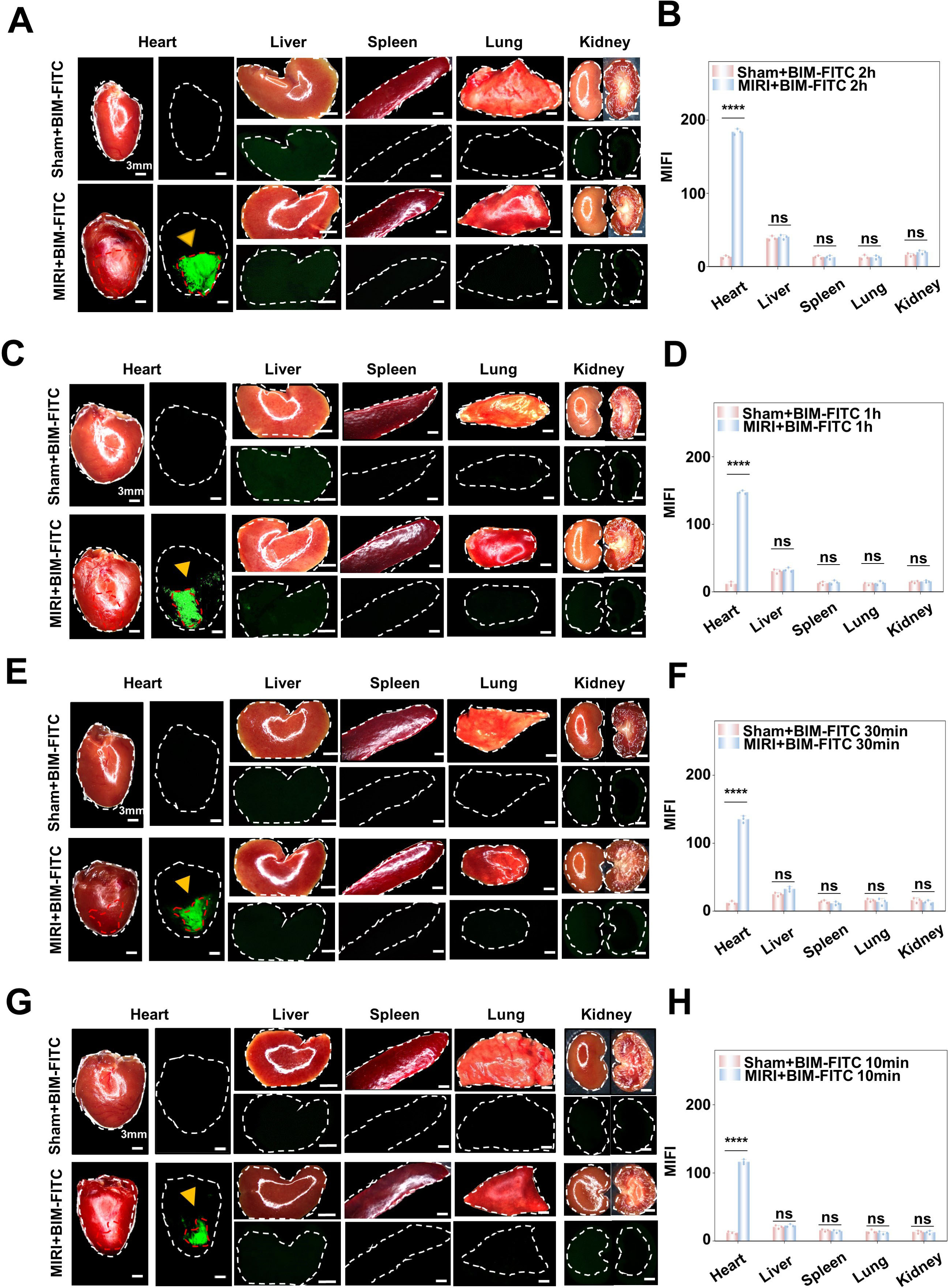
**

**Figure S3. Representative images of BIM-FITC fluorescence imaging and bright field in the hearts, livers, spleens, lungs and kidneys of Sham and MIRI rat at 10min, 30min, 1h and 2h after drug administration, as well as MFI statistics.** Representative images of Sham and MIRI rat at 2h after drug administration **(A)**, and MFI statistics **(B)**; Representative images of Sham and MIRI rat at 1h after drug administration **(C)**, and MFI statistics **(D)**; Representative images of Sham and MIRI rat at 30min after drug administration **(E)**, and MFI statistics **(F)**; Representative images of Sham and MIRI rat at 10min after drug administration **(G)**, and MFI statistics **(H)**; scale bar: 2mm. Data were expressed as mean ± SE. Statistical significance was performed by unpaired two-tailed Student’s t test. (n = 3, ns: *P* ＞ 0.05, *^****^P* < 0.0001)

***
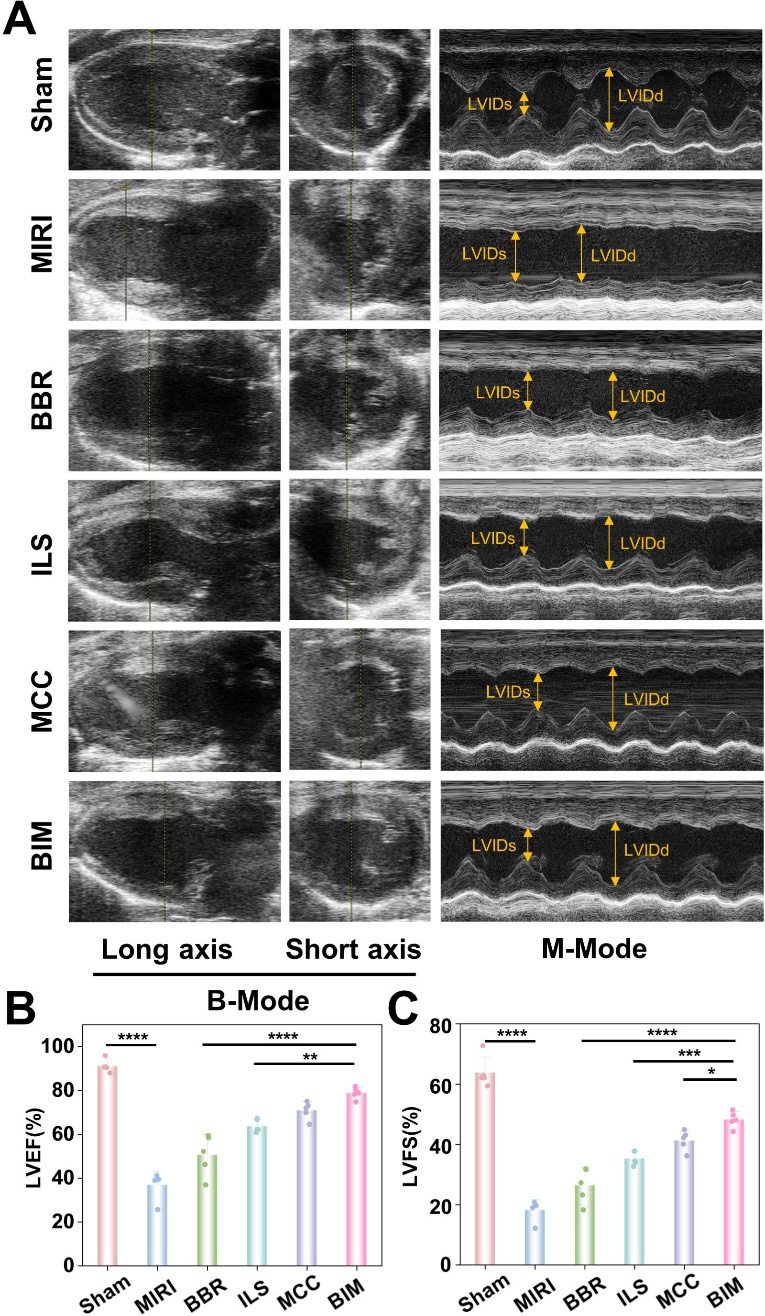
***

**Figure S4. Cardiac Function Assessment. (A)** Characteristic echocardiography image after treatment with at 24 h after MIRI and quantitative analysis of **(B)** LVEF and **(C)** LVFS (n = 5). Data were expressed as mean ± SE. Statistical significance was performed by one-way ANOVA with Tukey post hoc test. (n = 3, **P* < 0.05, ***P* < 0.01, ****P* < 0.001, *****P* < 0.0001, **P* < 0.05).


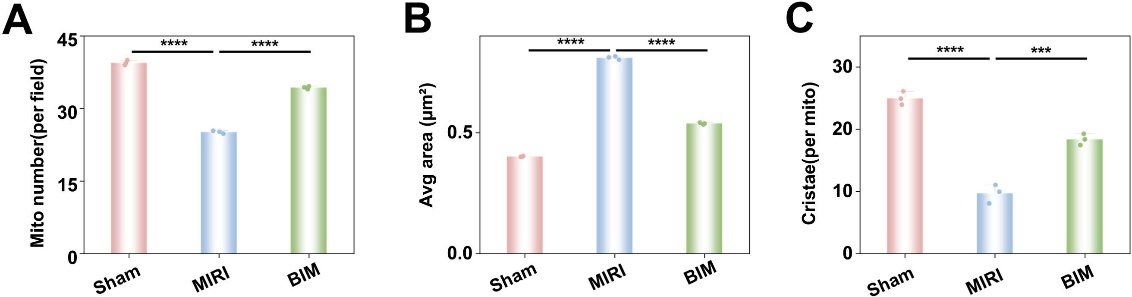


**Figure S5. Quantitative analysis of the mitochondrial number (A), average mitochondrial area (B), and cristae count (C) in TEM images of mitochondria in cardiac tissues from Sham, MIRI and BIM treated groups.** Data are expressed as mean ± SE. Statistical significance was performed by one-way ANOVA and Tukey post hoc test. (n = 3, ****P* < 0.001, *****P* < 0.0001).


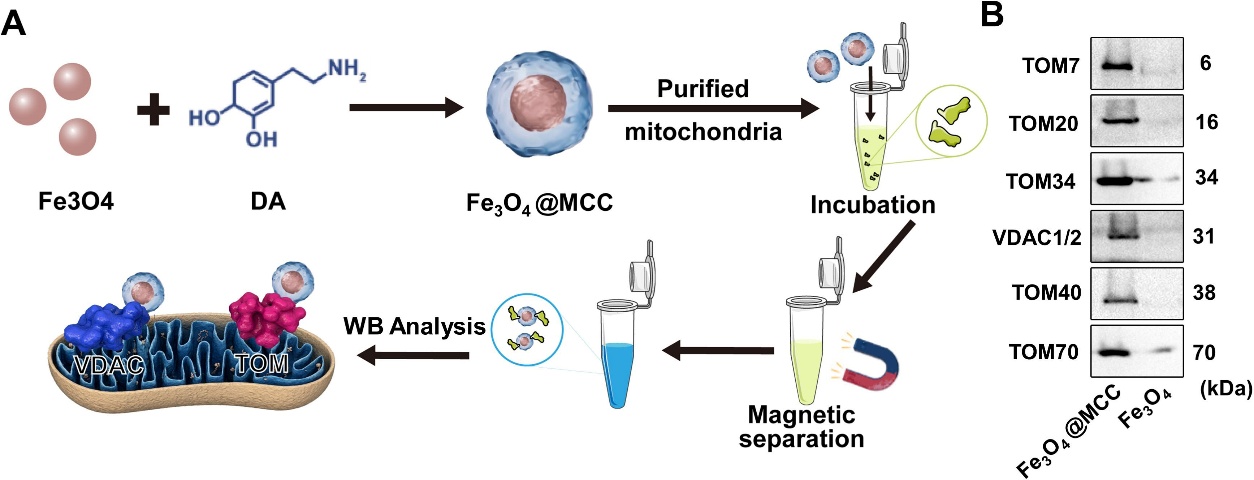


**Figure S6. MCC-mediated mitochondrial targeting.** (A) Brief diagram of the Fe_3_O_4_@MCC synthesis process, and the selective enrichment and identification of mitochondrial proteins by magnetic separation and WB analysis. (B) WB analysis of TOM7, TOM20, TOM34, VDAC1/2, TOM40, TOM70 adsorbed on Fe_3_O_4_ and Fe_3_O_4_@MCC.


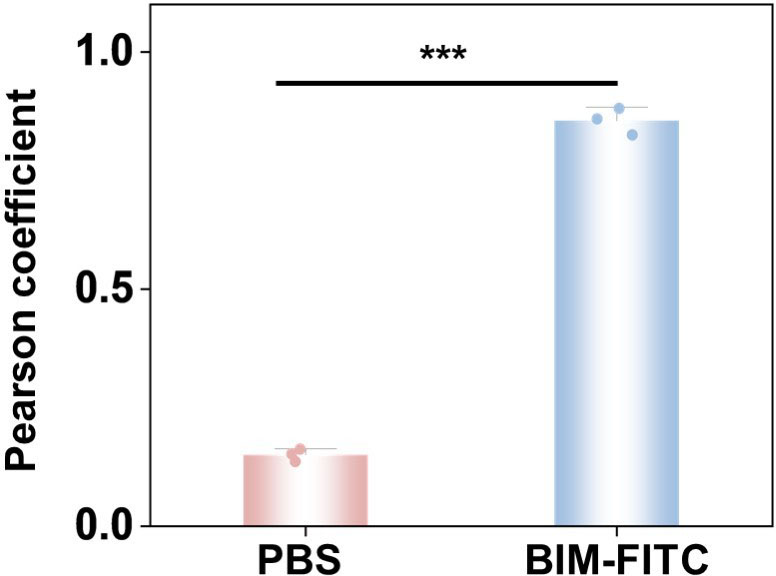


**Figure S7.** **Quantitative analysis of co-localization of BIM-FITC or PBS with H9c2 cell mitochondria for fluorescent staining.** Data are expressed as mean ± SE. Statistical significance was performed by one-way ANOVA and Tukey post hoc test. (n = 3, ****P* < 0.001).

**
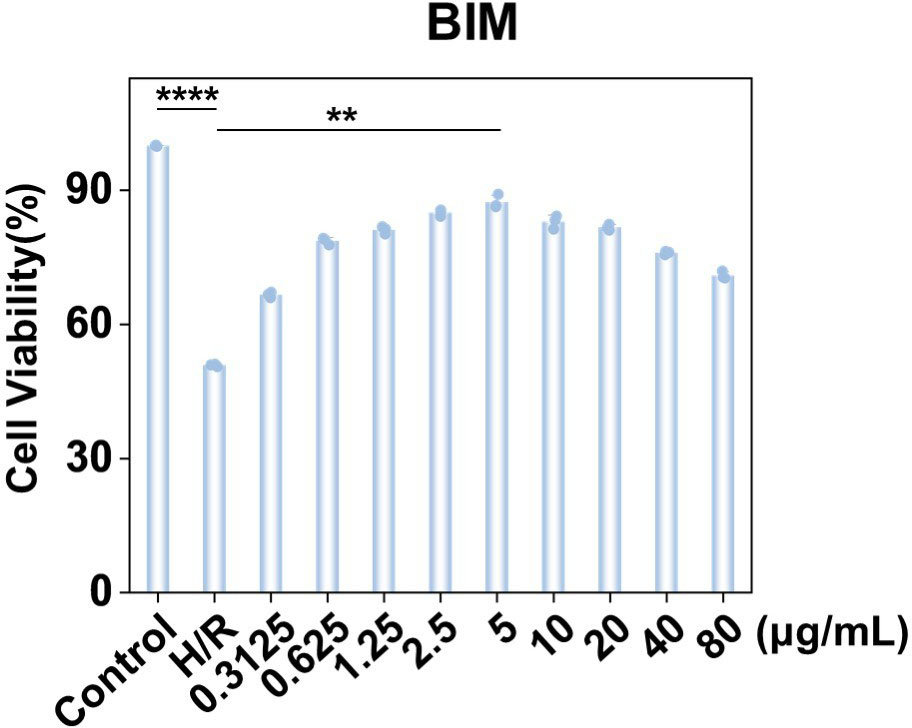
**

**Figure S8. Cell viability of H9c2 cells under H/R condition after treatment with different concentrations of BIM for 24 h. Cell viability results were detected by CCK8.** Data are expressed as mean ± SE. Statistical significance was performed by one-way ANOVA and Tukey post hoc test. (n = 6, ^**^*P* < 0.01, ^****^*P* < 0.0001).

***
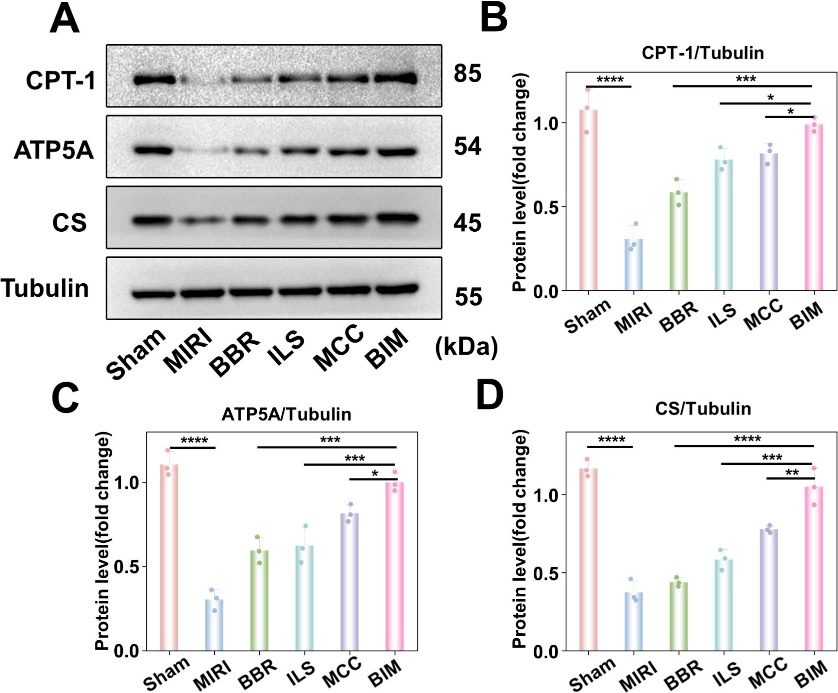
***

**Figure S9.** **Representative images and quantitative analysis of WB of mitochondrial function-related proteins in cardiac tissues.** Data were expressed as mean ± SE. Statistical significance was performed by one-way ANOVA with Tukey post hoc test. (n = 3, **P* < 0.05, ***P* < 0.01, ****P* < 0.001, *****P* < 0.0001, **P* < 0.05).

***
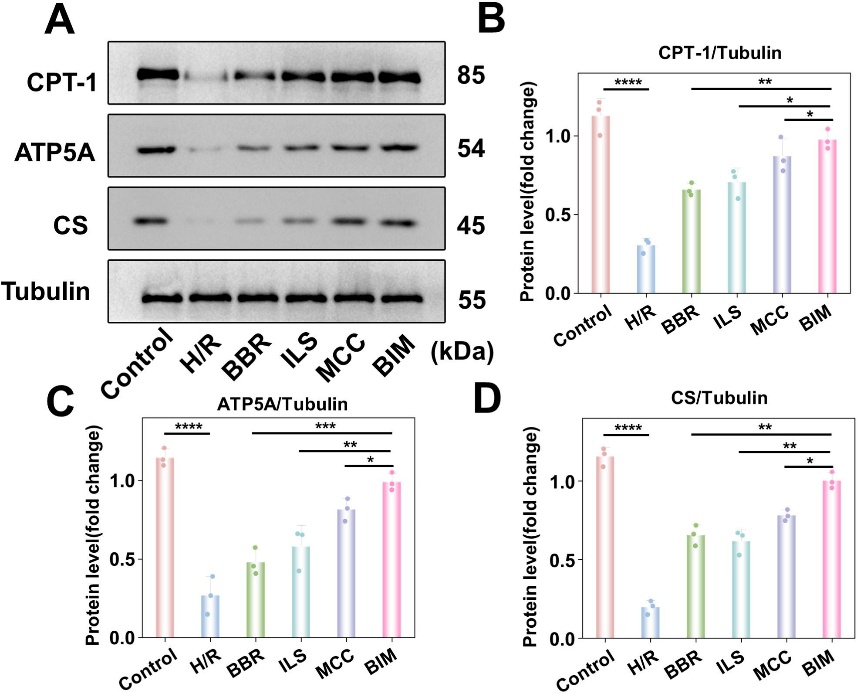
***

**Figure S10.** **Representative images and quantitative analysis of WB of mitochondrial function-related proteins in H9c2 cells.** Data were expressed as mean ± SE. Statistical significance was performed by one-way ANOVA with Tukey post hoc test. (n = 3, **P* < 0.05, ***P* < 0.01, ****P* < 0.001, *****P* < 0.0001, **P* < 0.05).


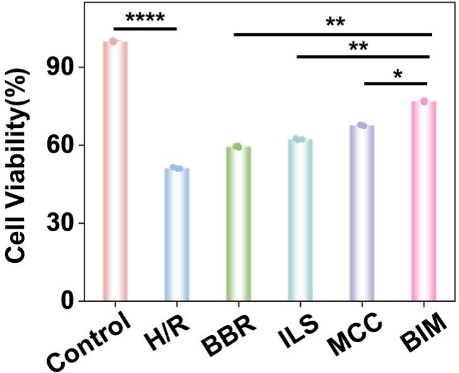


**Figure S11. Cell viability of H9c2 cells under different treatment.** Data were expressed as mean ± SE. Statistical significance was performed by one-way ANOVA with Tukey post hoc test. (n = 3, **P* < 0.05, ***P* < 0.01, *****P* < 0.0001, **P* < 0.05).


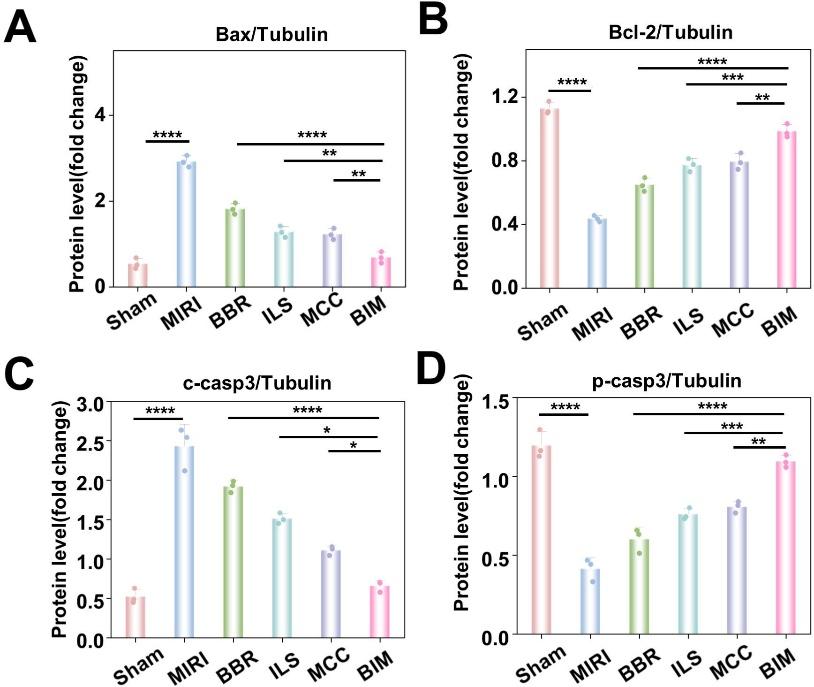


**Figure S12. Quantitative analysis of WB of Bax、Bcl-2、c-casp3 and p-casp3 in cardiac tissues.** Data were expressed as mean ± SE. Statistical significance was performed by one-way ANOVA with Tukey post hoc test. (n = 3, **P* < 0.05, ***P* < 0.01, ****P* < 0.001, *****P* < 0.0001, **P* < 0.05).


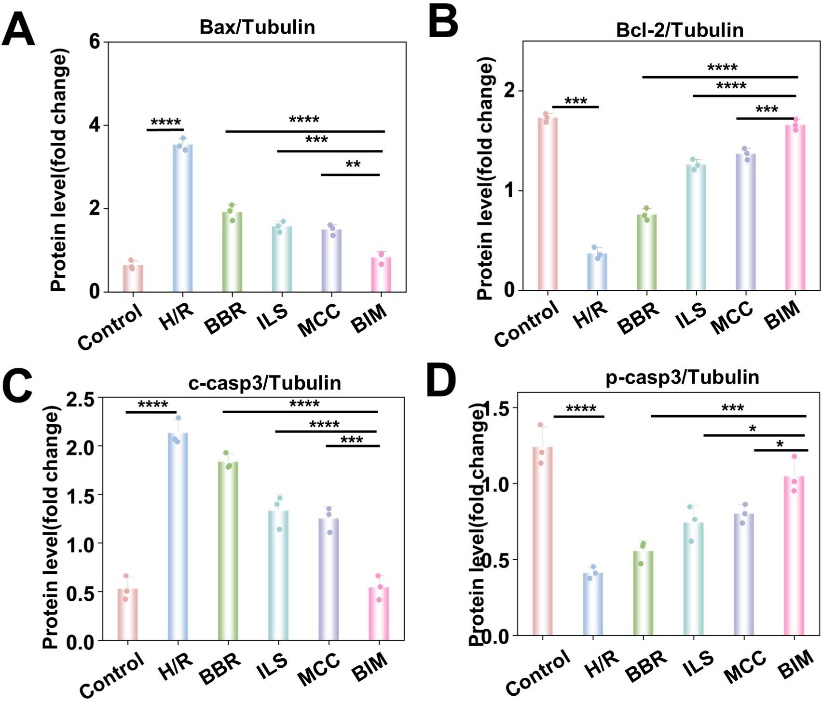


**Figure S13. Quantitative analysis of WB of Bax、Bcl-2、c-casp3 and p-casp3 in H9c2 cells.** Data were expressed as mean ± SE. Statistical significance was performed by one-way ANOVA with Tukey post hoc test. (n = 3, **P* < 0.05, ***P* < 0.01, ****P* < 0.001, *****P* < 0.0001, **P* < 0.05).


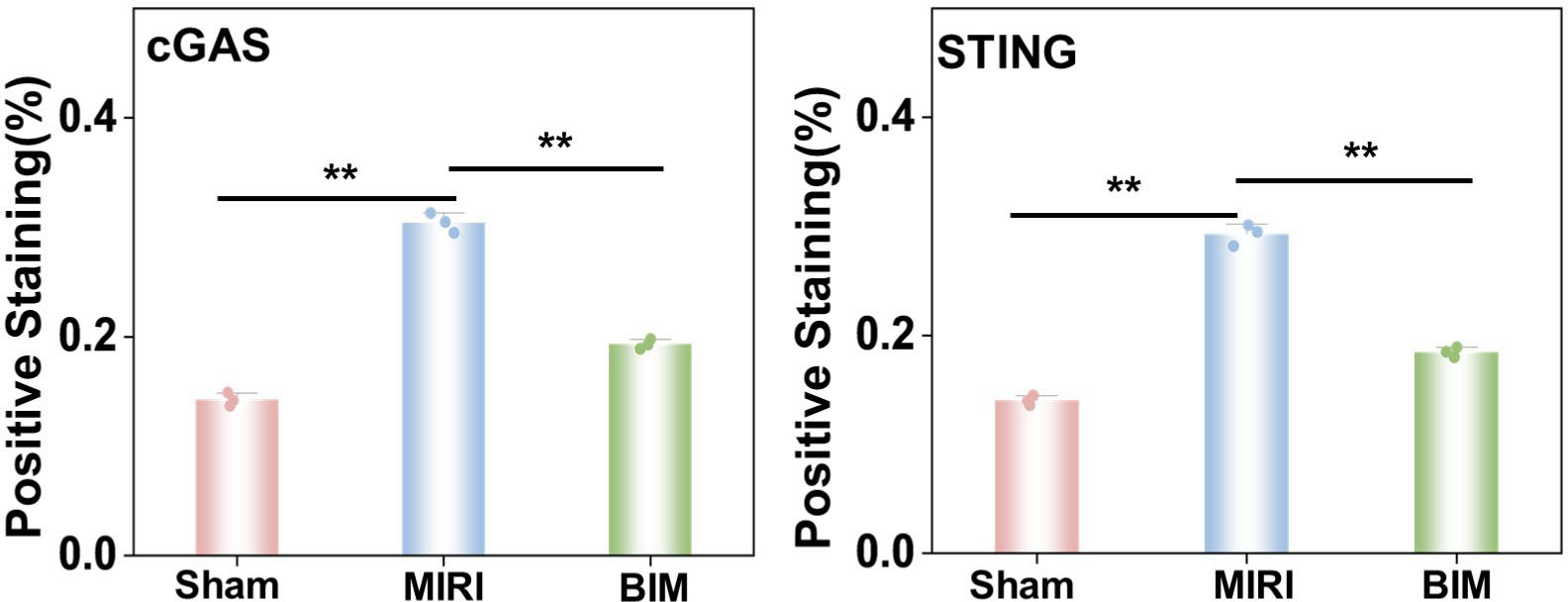


**Figure S14. Quantitative statistics of immunohistochemical staining of cGAS and STING in different groups of cardiac tissues and.** Data were expressed as mean ± SE. Statistical significance was performed by one-way ANOVA with Tukey post hoc test. (n = 3, *^**^P* < 0.01).


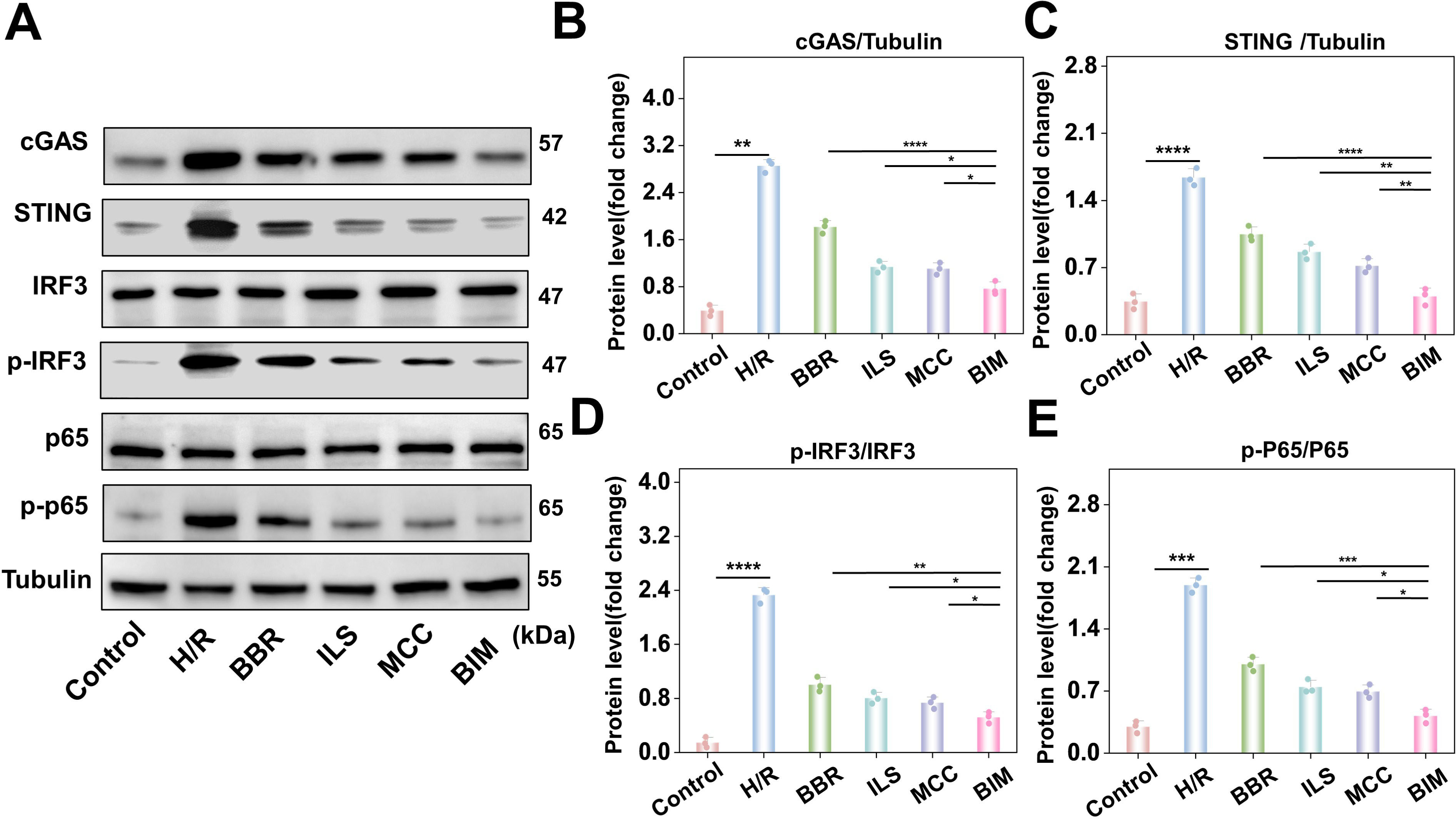


**Figure S15. Representative images and quantitative analysis of WB of cGAS–STING pathway-related proteins in H9c2 cells.** Data were expressed as mean ± SE. Statistical significance was performed by one-way ANOVA with Tukey post hoc test. (n = 3, *^*^P* < 0.05, *^**^P* < 0.01, *^***^P* < 0.001, *^****^P* < 0.0001).


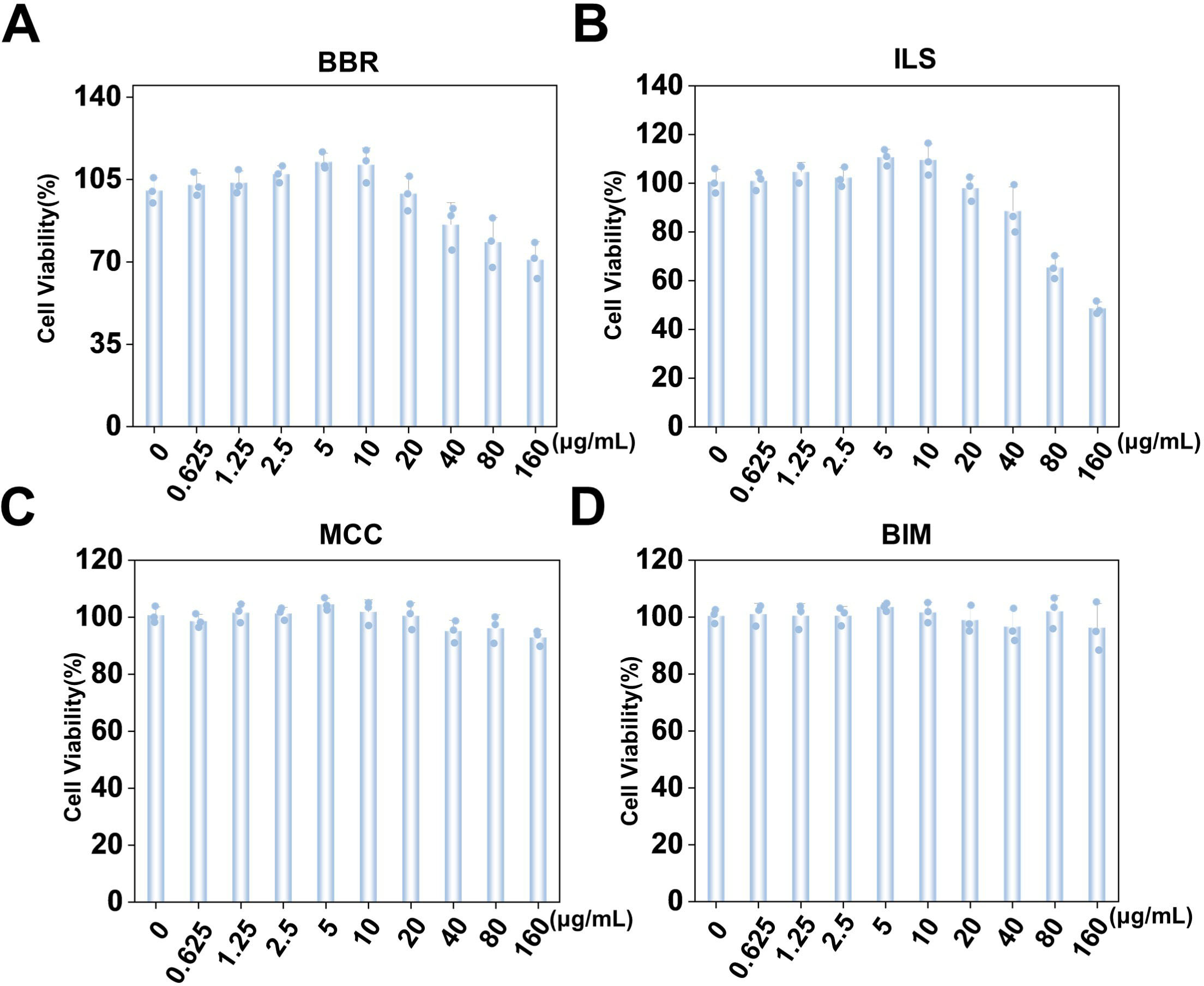


**Figure S16. Cell viability of H9c2 cells after treatment with different concentrations of BBR, ILS, MCC and BIM for 24 h. Results of cell viability detection by CCK8.**
